# Molecular basis of the short- and long-term osmoregulation capability in the euryhaline unicellular eukaryote *Paramecium calkinsi*

**DOI:** 10.1128/mbio.03032-25

**Published:** 2026-03-06

**Authors:** Jia Liu, Juan Yang, Xue Zhang, Rui Wang, Lei Yang, Huan Dou, Rebecca A. Zufall, Xiao Chen, Feng Gao

**Affiliations:** 1 Key Laboratory of Evolution & Marine Biodiversity (Ministry of Education) and Institute of Evolution & Marine Biodiversity, Ocean University of China 12591, Qingdao, China; 2 Marine College, Shandong University 601999 , Weihai, China; 3 Department of Biology and Biochemistry, University of Houston 14743, Houston, Texas, USA; 4 Laboratory for Marine Biology and Biotechnology, Qingdao Marine Science and Technology Center 554912 , Qingdao, China; Oregon State University, Corvallis, Oregon, USA

**Keywords:** *Paramecium calkinsi*, osmoregulation, euryhaline, genome, transcriptome

## Abstract

Euryhaline species exhibit remarkable osmoregulatory adaptability, yet their underlying molecular basis of osmoregulation—especially those governing short-term versus long-term adaptation—remains poorly understood. This study investigates these issues using the euryhaline unicellular eukaryote *Paramecium calkinsi*. Based on a *de novo* assembled high-quality genome, comparative genomic analysis across 17 species revealed an expansion of osmoregulatory genes, identifying 195 expanded gene families implicated in oxidoreductase activity, ion binding, and transmembrane transport. Transcriptomic analysis under varying salinity treatments revealed distinct molecular foundations that underpin transient and sustained adaptation to hyperosmotic and hypoosmotic stress. Under hyperosmotic conditions, cells exhibit a rapid activation of the membrane transport system in the short term, whereas long-term adaptation features enhanced ribosome biogenesis, chromatin remodeling, activation of transport systems, and metabolic downregulation to optimize energy use. For hypoosmotic stress, short-term adaptation is characterized by specific hydrolysis of intracellular substances, while long-term adaptation involves sustained oxidoreductase activation and enhanced vesicular/proton transport to maintain oxidative balance and cellular homeostasis. Moreover, we found that genes involved in signaling, transport, and protein stability pathways exhibit high rates of alternative splicing, suggesting a potential role of mRNA splicing in osmoregulation. Our findings provide molecular insights into osmoregulation in unicellular eukaryotes, highlighting their unique genetic responses to transient and sustained salinity stress and offering a valuable starting point for future functional studies.

**IMPORTANCE:**

Euryhaline species exhibit significant adaptability to different salinities. This study elucidates how a single-celled euryhaline eukaryote navigates both transient and sustained salinity shifts at the molecular level. Comparative genomic analysis revealed that this organism expanded 195 gene families involved in ion transport and stress response. Transcriptomic analysis revealed distinct molecular foundations that underpin its transient and sustained adaptation to salinity stress. For high salinity, it transiently activates membrane transport systems, while long-term adaptation focuses on reprogramming metabolism to optimize energy use. In response to low salinity, the short-term response involves hydrolyzing intracellular materials, followed by the long-term activation of protective mechanisms. Additionally, alternative splicing fine-tunes genes involved in signaling and transport. These findings reveal unique genetic and cellular adaptation to salinity fluctuations in unicellular eukaryotes and establish a valuable resource for future functional investigations.

## INTRODUCTION

Salinity constitutes one of the crucial limiting factors for aquatic organisms (1–3). The salinity range that organisms can tolerate is limited, primarily depending on their ability to regulate osmotic pressure (4). Organisms capable of thriving across a wide range of external salinity changes are termed euryhaline species (5), predominantly observed among multicellular organisms. The regulation of osmotic pressure in different organisms is complex and varied, with multiple mechanisms often working in concert (6, 7).

For multicellular organisms, the most common adaptation mechanism is structural specialization, such as gill filaments in fish that excrete NaCl (8) and stomata in plants that autonomously open and close (9). Additionally, hormonal regulation plays a crucial role, with cortisol, oxytocin, and growth hormone in animals (10, 11), and abscisic acid, gibberellins, and cytokinins in plants (12), all contributing to osmotic pressure regulation. At the cellular level, the basic adaptive strategy involves regulating ion transport and osmotic regulators, with the balance of Na^+^, K^+^, and H^+^ being essential for maintaining cellular homeostasis (13). Various organic substances also help maintain osmolality in living organisms. For example, proline, sugar alcohols, sorbitol, quaternary ammonium compounds, and α-amino nitrogen have been reported to be relevant to osmolality regulation in plants (14, 15), while sorbitol, glycine betaine, inositol, and taurine are reported in mammals (16).

Single-celled organisms, being directly exposed to their surrounding environment (17–19), exhibit high sensitivity to osmotic changes. This heightened responsiveness has spurred the evolution of diverse adaptive strategies to regulate and mitigate the impacts of varying osmotic pressures. These responses encompass the passive exchange of electrolytes, the active synthesis of compatible osmolytes (20), and the adaptation of the cell wall to endure significant hydrostatic pressures and so on (21, 22). Studies on halophilic ciliates and green algae have demonstrated that these organisms employ a salt-out strategy to maintain osmotic homeostasis, primarily by extruding excess salt ions while accumulating high concentrations of compatible solutes (23). An increasing number of studies have investigated various types of compatible solutes in unicellular organisms. Among these solutes, glycerol has been well established as a compatible osmolyte in various fungi (23) and green algal species (24). In halophilic ciliates *Halocafeteria seosinensis* and *Pharyngomonas kirbyi*, osmotic stress adaptation has been linked to the biosynthesis and transport of compatible solutes such as 5-hydroxyinositol and myo-inositol (25). Previous research has demonstrated that a variety of unicellular organisms are capable of adapting to euryhaline conditions (26, 27), but the underlying molecular regulatory mechanisms governing this adaptation are still largely unknown.

Ciliates, as single-celled eukaryotic organisms lacking cellular walls, are widely distributed in diverse habitats including soil, swamps, lakes, wetlands, and marine environments (28–32). As a well-known group of ciliates, *Paramecium* species are generally found in freshwater (33), but *Paramecium calkinsi* is renowned for its exceptional ability to thrive in a wide range of salinity conditions (34, 35). Therefore, *P. calkinsi* offers an intriguing model for investigating cellular adaptation under osmotic pressure. Although there are studies focusing on the ion transport enzymes and free amino acids during osmoregulation in *P. calkinsi* (36, 37), the molecular mechanism is still unknown, especially how cells respond to instant and long-term osmotic pressures.

In the current work, we sequenced and assembled the genome of *P. calkinsi* using both PacBio long-read sequencing and short-read sequencing. To investigate both the instant and long-term osmoregulation mechanisms of *P. calkinsi*, we designed experiments with varying osmotic pressure levels, treating the cells over both short-term and long-term durations, and subsequently acquired their transcriptome profiles. Comprehensive analyses allow us to elucidate the regulatory mechanisms governing osmoregulation in *P. calkinsi* at both genomic and transcriptomic levels for the first time. Leveraging gene manipulation techniques, we have verified pivotal genes and metabolic pathways associated with osmotic pressure adaptation in *P. calkinsi*. These findings establish a robust foundation for unveiling the osmoregulation strategies in single-celled organisms.

## RESULTS

### Assembly and features of *Paramecium calkinsi* macronuclear genome

**Fig 1:**
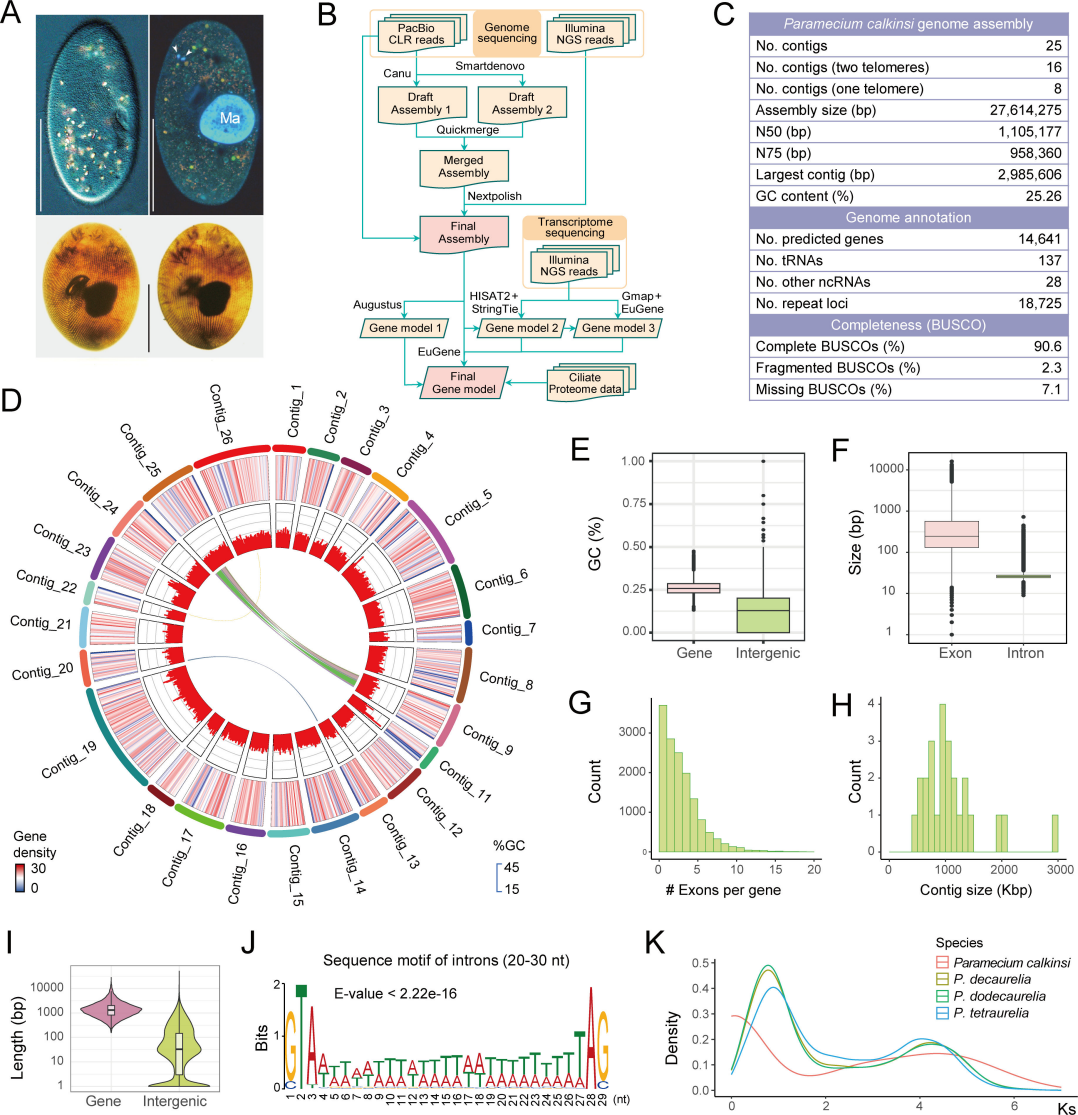
Sequencing and assembly of the somatic genome of *Paramecium calkinsi*. (**A**) Photo of *P. calkinsi* under a microscope *in vivo* (top left), after fluorescence staining by Hoechst 33342 and acridine orange (top right), and after silver carbonate impregnation (bottom left and bottom right for ventral and dorsal views, respectively). Scale bars = 50 µm. “Ma” and arrows denote the macronucleus and the micronuclei of *P. calkinsi*, respectively. (**B**) Schematic diagram of the strategy for the genome assembly and annotation. (**C**) Summary of the genome assembly features. (**D**) Circos plot showing the gene density (outer layer) and GC content (inner layer) of the *P. calkinsi* genome in 30 kb windows. The central links denote synteny groups across different chromosomes. (**E**) GC content comparison between gene and intergenic regions. (**F**) Size distribution of exons and introns. (**G**) Distribution of the numbers of exons in each gene. (**H**) Distribution of the contig sizes in the genome assembly. (**I**) Length of genes and intergenic regions. (**J**) Sequence motif of introns with lengths between 20 and 30 nt. (**K**) Ks distribution curves indicating the whole-genome duplication events for *P. calkinsi* (red), *P. decaurelia* (yellow), *P. dodecaurelia* (green), and *P. tetraurelia* (blue).

In the culture process, we assessed the osmotic tolerance of *P. calkinsi*. The cells exhibited an extraordinary capability to survive in salinities ranging from 0 to 60‰. Specifically, cells transferred from 30‰ to 15‰ or 45‰ maintained the typical morphology, as shown in Fig. 1A. Under more extreme conditions (0‰–5‰ and 60‰), although the cells initially exhibited significant deformation—swelling at low salinities and flattening at 60‰—they were able to recover their normal shape after adaptation. Moreover, the cells remained viable and proliferative throughout these transitions. Meanwhile, after mild starvation, conjugation could effectively occur under these varying pressures, except at 60‰. To better understand the mechanistic and evolutionary basis of its exceptional osmotic tolerance, we first characterized the macronuclear genome of *P. calkinsi*, combining both PacBio continuous long read (CLR) sequencing and short-read sequencing techniques (Fig. 1B; Fig. S1, Table S1). The MAC genome of *P. calkinsi*, assembled from PacBio CLR using Canu and SMARTdenovo, comprises 25 contigs with a total size of 27.6 million base pairs (Mb), including 16 complete chromosomes with both telomeres, eight contigs containing a single telomere, and one contig lacking telomeres (Fig. 1C). The assembled contigs range in size from 500 kilobase pairs (Kb) to 3 Mb, with an N50 value of 1.1 Mb.

We predicted 14,641 protein-coding genes from the assembled genome and annotated them by InterProScan and BLAST using Pfam and ciliate protein sequences as reference databases, respectively (Fig. 1C; Fig. S2A). The mean gene density on the chromosomes is ~16.6 genes per 30-kilobase window, and the global GC content is 25.26% (Fig. 1D). The coding regions and the intergenic regions have GC content of 26.06% and 18.14%, respectively (Fig. 1E). The length of chromosomes peaks at 1 Mb (Fig. 1H). The mean length of coding and intergenic regions is 1,712 bp and 174 bp, respectively (Fig. 1I). Notably, most genes contain fewer than 10 exons in *P. calkinsi*, with an average exon length of 474 bp (Fig. 1F and G). The introns are predominantly small in size (20 bp–30 bp, mean size of 27.8 bp) with a canonical 5′-GT-AG-3′ boundary motif at splice junctions (Fig. 1J). BLAST analysis of *P. calkinsi* proteins revealed *Paramecium* species as the exclusive top hits (Fig. S2B). The BUSCO analysis that evaluates the genome assembly completeness based on the current gene model showed that complete and fragmented BUSCOs are 90.6% and 2.3%, respectively (Fig. 1C).

We also predicted 137 tRNAs and 28 other ncRNAs (Fig. 1C). The tRNAs cover anticodons for all 20 amino acids. The codon usage frequency plot reveals that *P. calkinsi* exhibits remarkable diversity in anticodon usage, suggesting an adaptive capacity to flexibly respond to varied codon contexts (Fig. S2E). Notably, amino acids such as methionine (Met), asparagine (Asn), aspartic acid (Asp), cysteine (Cys), histidine (His), phenylalanine (Phe), tryptophan (Trp), and tyrosine (Tyr) codons are each represented by a single anticodon. Met displays an extreme bias, with the CAT anticodon appearing at a high frequency (nine instances). The stop codon usage analysis for the genes that are highly similar to other ciliates revealed an obvious reassignment of the stop codons UAA and UAG to codons encoding amino acids (Fig. S2D and E). Besides, 28 other ncRNAs include snRNA, snoRNA, rRNA, SRP RNA, and ribozyme-related RNA (Table S2).

In addition, 18,725 repeat loci were identified. The repeat loci are predominantly composed of simple repeats (15,528; 82.9%), while low-complexity repeats constitute a minor fraction (3,197; 17.1%). No transposable elements (LINE/SINE/LTR) or retrotransposons were detected, indicating a uniquely streamlined repeat architecture (Fig. S2C).

Based on the cDNA sequences of the annotated genes, whole-genome duplication (WGD) analysis suggested that there was only one recent WGD event in *P. calkinsi*, rather than two recent WGD events in other *Paramecium* species including *P. tetraurelia*, *P. decaurelia*, and *P. dodecaurelia* (38–40) (Fig. 1K).

### Differential gene expression analysis under different salinity treatments

**Fig 2:**
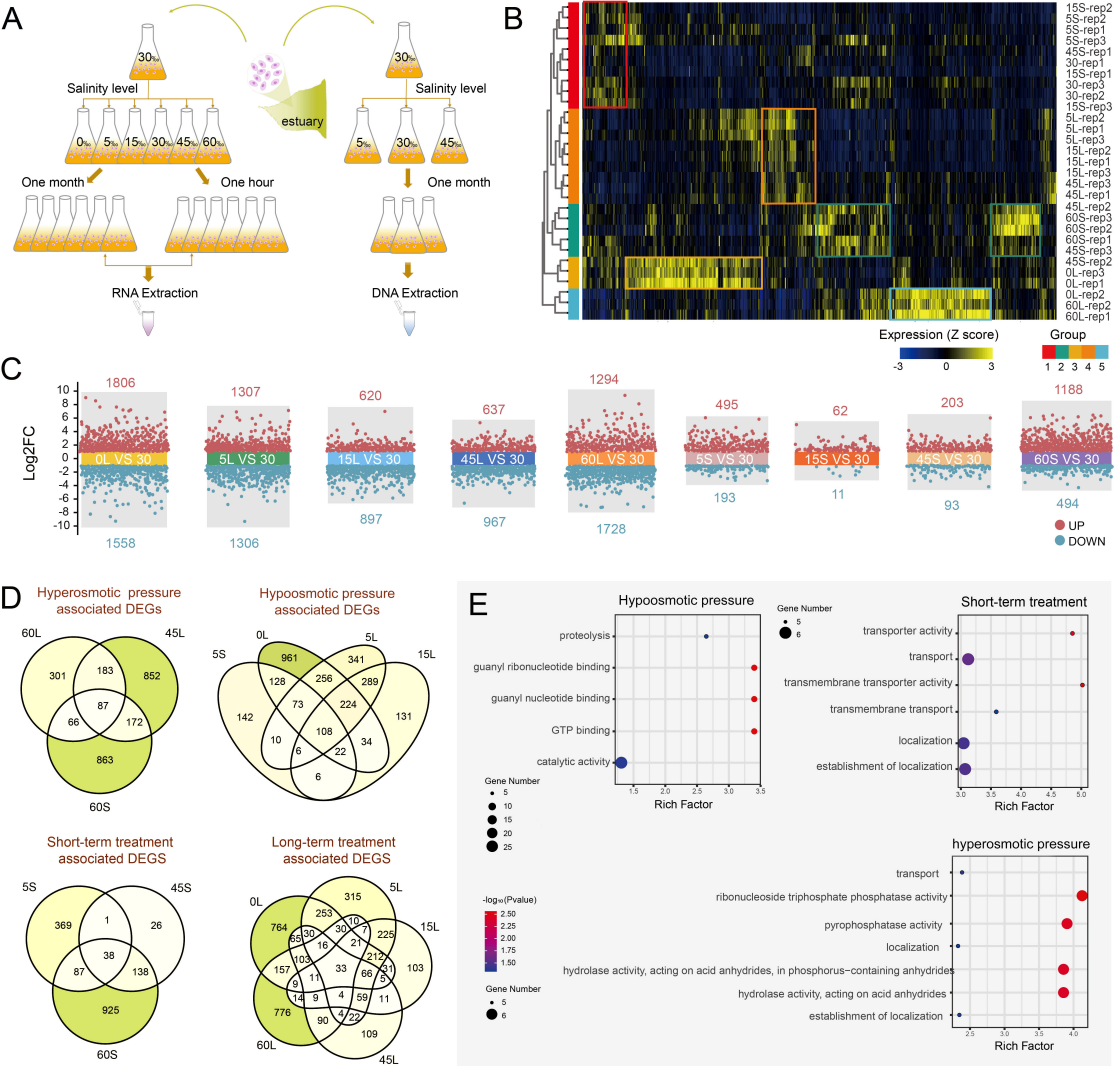
Transcriptome profile changes under different osmotic pressures. (**A**) Schematic diagram of the experimental design for cell treatment under different osmotic pressures. (**B**) Heatmap of differential gene expression. “S” represents short-term treatment and “L” represents long-term treatment. In the differential expression analysis, the same 30‰ control group (30) was used as the reference for both short-term and long-term treatments. (**C**) Differential gene expression analysis showing up- and downregulated genes under short- or long-term osmotic pressures. Upregulated genes are shown in red (log2FC > 1, adjusted *P*-value <0.1) while downregulated genes are shown in blue (log2FC < −1, adjusted *P*-value <0.1). (**D**) Venn diagram showing the overlaps between DEGs in cells under either hypoosmotic or hyperosmotic pressure levels and short- or long-term treatment. (**E**) GO enrichment analysis of shared genes under hypoosmotic, hyperosmotic, and short-term treatments.

To reveal the gene expression changes in response to varying osmotic pressures, we obtained and analyzed transcriptomic data from *P. calkinsi* cells cultured under different osmotic pressures (*n* = 3 for each group; Fig. 2A). The cells were transferred from a salinity of 30‰ to the target salinity medium (0‰, 5‰, 15‰, 30‰, 45‰, and 60‰), treated for 1 h (short-term treatments, except for 0‰) or at least 1 month (long-term treatments), respectively. Transcriptome analysis indicated that *P. calkinsi* cells under different osmotic pressures, upon either short- or long-term treatments, showed diverse transcriptomic profiles (Fig. 2B; Fig. S3A).

Differential gene expression analysis under long-term salinity stress, with 30‰ as control, revealed diverse transcriptional responses across the salinity gradient (*n* = 3 for each group; Fig. 2C). Notably, when cells were transferred from 30‰ to both lower (0‰, 5‰, and 15‰) and higher (45‰ and 60‰) osmotic pressures, greater osmotic shifts induced more pronounced transcriptional changes.

Cells under the 0‰ salinity long-term treatment exhibited the most pronounced stress response, characterized by 1,806 upregulated and 1,558 downregulated genes. Gene Ontology (GO) enrichment analysis of these upregulated genes revealed significant enrichment of pathways such as oxidoreductase activity acting on the CH-CH group of donors, proton antiporter activity, monooxygenase activity, membrane protein complex, and coated membrane, suggesting the activation of osmotic homeostasis and xenobiotic detoxification mechanisms (Fig. S3B through F). For cells under 5‰ salinity long-term treatment, 1,307 genes were upregulated while 1,306 genes were downregulated. The upregulated genes showed similar pathway enrichments to those at 0‰ salinity, including oxidoreductase activity, monooxygenase activity, and coated membrane. Additionally, functional enrichments were identified in vesicle-mediated transport and regulation of response to stimulus (Fig. S3B, Table S3). For cells under 15‰ salinity long-term treatment, the number of upregulated genes decreased to 620, while downregulated genes were 897. The differentially expressed genes were primarily enriched in protein transport, oxidoreductase activity, intracellular protein transport, and catalytic activity pathways (Fig. S3C, Table S3).

For cells under 45‰ salinity long-term treatment, the number of differentially expressed genes (637 upregulated and 967 downregulated) was similar to those in 15‰ salinity. However, their functional enrichment results differed significantly, with upregulated genes being enriched in transcriptional control machinery (including rRNA processing, ribosome biogenesis, and chromatin remodeling). This enrichment profile suggested a coordinated response to maintain proteostasis and to optimize energy allocation for gene expression, implying distinct regulatory mechanisms under hyperosmotic versus hypoosmotic conditions (Fig. S3E, Table S3).

Notably, hyperosmotic stress induced by 60‰ salinity long-term treatment triggered substantial transcriptomic changes (1,728 upregulated and 1,294 downregulated genes). GO enrichment analysis of these upregulated genes revealed significant enrichment in ATPase-coupled transmembrane transporter activity, ABC-type transporter activity, and primary active transporter activity, while downregulated genes were enriched in lipid catabolic processes, carbohydrate metabolic processes, and proteolysis. This enrichment profile revealed a coordinated cellular adaptation strategy to hyperosmotic stress, characterized by upregulation of ion transport systems coupled with downregulation of metabolic pathways (Fig. S3F, Table S3).

For short-term treatments, fewer genes exhibited differential expression compared to the long-term treatments of the corresponding salinity (Fig. 2C). Among these, the upregulated genes were primarily enriched in pathways related to transmembrane transport, localization, membrane function, and transporter activity (*P* < 0.05*,* Fig. 2E).

To investigate the gene regulatory features of *P. calkinsi* under hyperosmotic pressure, hypoosmotic pressure, as well as short-term and long-term treatment conditions, we analyzed the upregulated genes shared among the four distinct treatment groups. Owing to the extremely low number of differential genes in the short-term treatments of 15‰ and 45‰ salinity groups, comparisons involving these groups were excluded from the corresponding analyses.

For the hyperosmotic pressure group (60‰ short-term, 45‰ long-term, and 60‰ long-term treatments), there were 87 shared genes. Functional enrichment analysis demonstrated that these genes were enriched in pathways associated with transport, ribonucleoside triphosphate phosphatase activity, pyrophosphatase activity, localization, hydrolase activity (acting on acid anhydrides within phosphorus-containing anhydrides), and the establishment of localization (Fig. 2D and E).

In the hypoosmotic pressure group (5‰ short-term, 0‰ long-term, 5‰ long-term, and 15‰ long-term treatments), 108 shared genes were identified. Their functions were enriched in pathways such as proteolysis, guanyl ribonucleotide binding, guanyl nucleotide binding, GTP binding, and catalytic activity (Fig. 2D and E).

For the short-term treatment group (5‰, 45‰, and 60‰), 38 shared genes were detected. Functional enrichment indicated that these genes were concentrated in pathways like transporter activity, transport, transmembrane transporter activity, transmembrane transport, as well as localization and the establishment of localization (Fig. 2D and E). Regarding the long-term treatment group (0‰, 5‰, 15‰, 45‰, and 60‰), although there were 33 shared genes, no pathways were significantly enriched in the functional enrichment analysis (Fig. 2D and E).

### Differential gene expression dynamics and gene co-expression network under different salinity treatments

**Fig 3:**
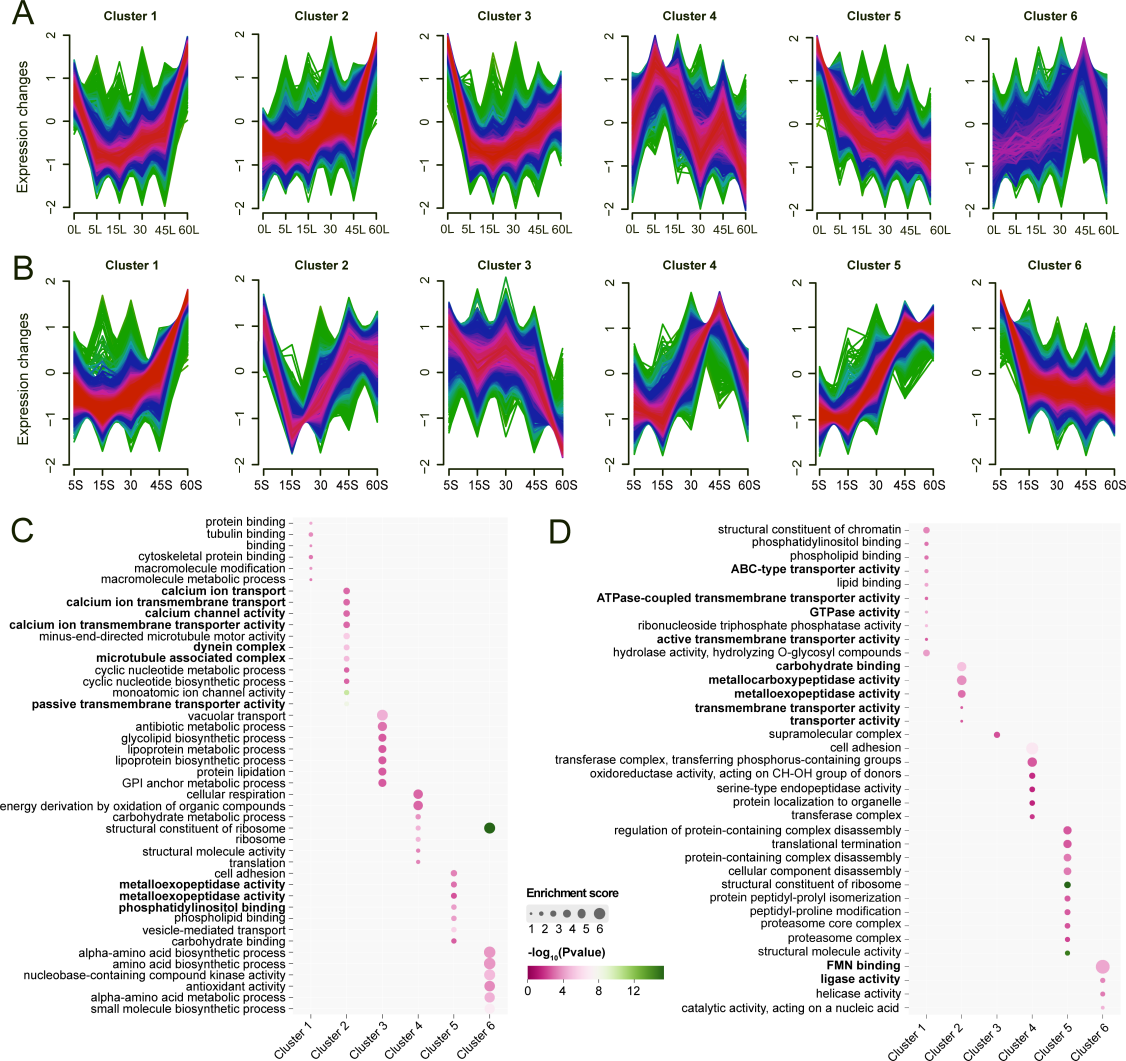
Differential gene expression dynamics and gene co-expression network under osmotic pressures. (**A**) Expression modulation across six gradients in long-term treatment (L). (**B**) Expression modulation across five gradients in short-term treatment (S). (**C**) Pathway enrichment analysis for each expression modulation under long-term treatment. (**D**) Pathway enrichment analysis for each expression modulation under short-term treatment.

To investigate the dynamics of gene expression under different salinity treatments, we classified the genes into six clusters according to their expression profiles and performed GO enrichment analysis for each cluster (Fig. 3). In the long-term salinity exposure (Fig. 3A and C), the gene expression in clusters 1 and 3 exhibited an asymmetric V-pattern (expression plunged sharply from 0‰ to a trough at 30‰, then partially increased toward 60‰). Genes in cluster 1 are enriched in protein binding, tubulin binding, and cytoskeletal protein binding, while genes in cluster 3 are enriched in GPI anchor metabolic process, vacuolar transport, lipoprotein metabolic process, etc. Cluster 2 gene expression exhibited sustained upregulation in response to increasing salinity. These genes are enriched in calcium ion transport, calcium ion transmembrane transport, minus-end-directed microtubule motor activity, and passive transmembrane transporter activity. Genes in cluster 4, enriched in cellular respiration, carbohydrate metabolic processes, structural constituents of ribosomes, and translation, exhibited significant upregulation at 5‰ salinity, followed by a fluctuating downward trend across higher salinities. The gene expression in cluster 5 showed an opposite trend to that of cluster 2, exhibiting a continuous decrease with increasing salinity. These genes are enriched in cell adhesion, metalloexopeptidase activity, and vesicle-mediated transport. Genes in cluster 6, enriched in alpha-amino acid biosynthetic process, amino acid biosynthetic process, and antioxidant activity, demonstrated a progressive upward trend with increasing salinity but decreased significantly at 60‰.

In the short-term treatments (Fig. 3B and D), gene expression in cluster 1 showed stable expression under low salinities but gradually increased under high salinities. These genes are enriched in phospholipid binding, ABC-type transporter activity, lipid binding, ATPase-coupled transmembrane transporter activity, GTPase activity, and hydrolase activity. Genes in cluster 2—functionally enriched in carbohydrate binding, transporter activity, and metalloexopeptidase activity—reached peak expression at 5‰ salinity, sharply declined at 15‰, and then gradually recovered with salinity increases. The gene expression in clusters 3 and 6 displayed overall declining trends with increasing salinity, functionally enriched in FMN binding, ligase activity, catalytic activity, and so on. Genes in clusters 4 and 5 demonstrated progressive upregulation with increasing salinity, yet diverged markedly at 60‰—cluster 4 exhibited a sharp decline while cluster 5 maintained ascent. Functionally, genes in cluster 4 centered on cell adhesion, organelle-targeted protein localization, and enzymatic catalysis (serine endopeptidases, CH-OH oxidoreductases, phosphorus-transfer transferases). Conversely, genes in cluster 5 prioritized macromolecular complex dynamics, including ribosomal structural integrity, proteasome assembly, translational termination, and regulated disassembly of protein complexes.

To identify key modules or genes associated with osmoregulation, we performed weighted gene co-expression network analysis (WGCNA) for cells cultured under different osmotic pressures upon long-term treatment (Fig. S4A). The result showed three strong gene co-expression modules that may play a synergistic role in response to osmotic pressure changes. The largest one consisted of genes induced by hyperosmotic pressure of 60‰, while the other two modules were associated with genes induced by hypoosmotic pressures (0‰ and 5‰). Pathway enrichment analysis revealed that both the hyperosmotic and hypoosmotic pressure-induced modules participated in hydrolase activity, but with different focuses on acid anhydrides and O-glycosyl compounds, respectively. In addition, ATP-dependent activities and GTP binding were also involved in cellular response to hyperosmotic and hypoosmotic pressures, respectively (Fig. S4B and C, Table S4).

### Genomic changes associated with osmoregulation

**Fig 4:**
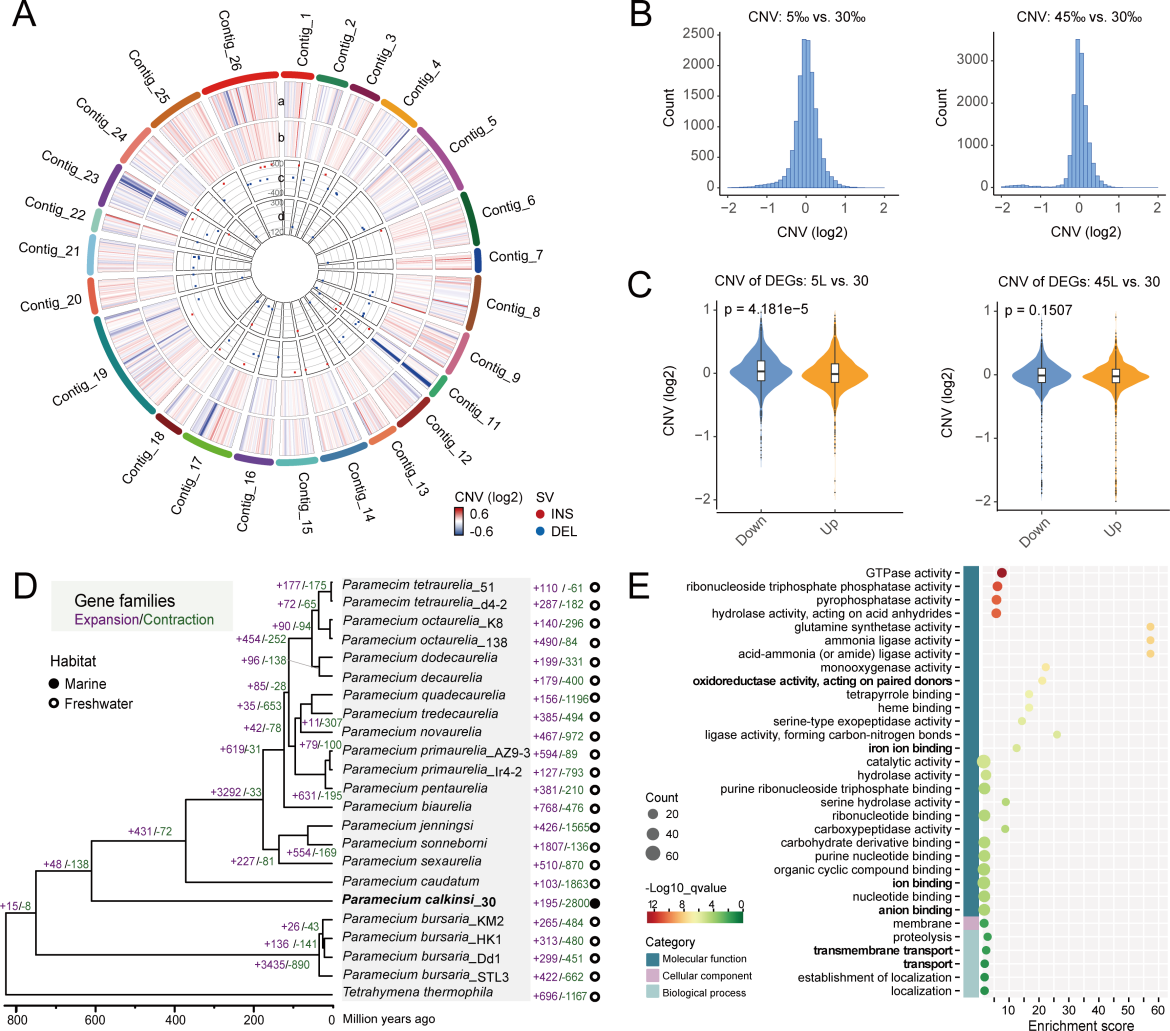
Genomic changes associated with osmotic pressure adaptation. (**A**) Circos plot showing the copy number variations (CNVs) in 30 Kb windows (outer layers), the structural variations (SVs, inner layers) in cells under hypoosmotic (5‰) and hyperosmotic pressures (45‰) under long-term treatment, using normal osmotic pressure (30‰) as control. “a–d” denote the tracks for CNV (5‰), CNV (45‰), SV (5‰), and SV (45‰), respectively. The color scales denote the fold change (log2) of CNVs. The scales of SVs denote the sizes of inserted or deleted fragments. (**B**) Distribution of gene numbers with different CNVs in cells under hypoosmotic (5‰, left) and hyperosmotic pressures (45‰, right) under long-term treatment, using normal osmotic pressure (30‰) as control. (**C**) The correlation between gene copy number variation and expression level. (**D**) Gene family evolution analysis for *Paramecium* species, using *Tetrahymena thermophila* as an outgroup. (**E**) Pathway enrichment analysis using the GO database for gene families expanded in *P. calkinsi*.

To investigate whether there is a genomic-level response to osmotic pressures in *P. calkinsi*, we sequenced and compared its MAC genomes under different osmotic pressures. Cells were transferred from a salinity of 30‰ to the target salinity (5‰, 30‰, and 45‰), treated for at least one month before DNA extraction and high-throughput sequencing. We also tried the treatment under 60‰. However, cells were in poor physiological condition (e.g., significantly inhibited growth and morphological abnormalities), resulting in failure to obtain high-quality genomic DNA for sequencing. By analyzing the whole-genome sequencing data, we detected structural variations and gene copy number variations in the MAC genome of *P. calkinsi* (Fig. 4A).

Compared to cells under a salinity of 30‰, which is the same as the ecosystem niche where *P. calkinsi* inhabits, hypoosmotic pressure (5‰) treatment induced more structural variations, especially deletions, than hyperosmotic pressure treatment (45‰) (Fig. 4A). Besides, both the hypoosmotic (5‰) and hyperosmotic pressures (45‰) had a strong impact on gene copy number variations at similar genomic loci (Fig. 4A and B). We detected 120 and 83 genes with copy number variations under hypoosmotic (5‰) and hyperosmotic (45‰) osmotic pressures, respectively, and 17 genes were shared by these treatments (Fig. S5D). Then we checked whether these genes exhibiting copy number variations showed differential expression under the corresponding salinity treatments. Though there was no obvious global correlation between gene copy number variations and expression changes under either hypoosmotic or hyperosmotic pressures (Fig. 4C; Fig. S5), positive correlation between gene copy number and expression levels was observed in specific genes (Fig. S5B and C, Table S5). For example, under the 5‰ osmotic pressure treatment, 24 genes showed coordinated increases in both expression levels and copy numbers, while 25 genes decreased expression corresponding to copy number reduction. Under the 45‰ pressure treatment, the expression levels of 3 genes were upregulated with copy number gains, whereas the expression levels of 38 genes were downregulated as their copy number decreased.

Changes in gene copy number are unlikely due to effects of sexual reproduction because all treatments were conducted using vegetative cells derived from a monoclonal cell line (no sexual process was observed) and maintained under constant nutrient-replete conditions, which are unlikely to induce sexual reproduction. Even if we cannot entirely rule out the possibility of a minuscule fraction of cells undergoing conjugation under stress, the data from this small number of sexual cells will likely be undetectable compared to the large number of vegetative cells. Therefore, the observed copy number variation events are most likely due to changes in macronuclear (MAC) chromosome copy number during vegetative reproduction. The model of chromosomal drift via unequal MAC division and selection has been studied in the ciliates *Fabrea salina* (41), *Oxytricha trifallax* (42), and *Halteria grandinella* (43). This model could explain our data if genes on the same MAC chromosome exhibit consistent copy number changes (all increasing or all decreasing) and could explain the lack of an overall correlation between gene copy number variations and their transcriptional expression levels. However, we observed concurrent copy number gains and losses of different genes within individual chromosomes. This pattern instead resembles findings in *Tetrahymena*, where MAC recombination underlies the expansion of metallothionein genes in response to metal stress (44). This suggests that multiple, non-mutually exclusive mechanisms may concurrently underlie MAC copy number variation in response to diverse environmental pressures.

To better understand the evolutionary strategy of *P. calkinsi* for osmoregulation, we identified orthologous protein sequences among 16 *Paramecium* species (22 strains in total, including *P. calkinsi*), as well as *Tetrahymena thermophila* (as an outgroup) and performed gene family evolution analysis (Fig. 4D). Based on the MCMC diagnostics, the divergence time estimates are well-supported, with all parameters exhibiting effective sample sizes substantially above 200, indicating reliable convergence and robust posterior inference for the phylogeny (Table S6). In the phylogenomic trees, *P. bursaria* strains first diverged from other *Paramecium* species, followed by *P. calkinsi*. Based on the estimated divergence time (662–934 million years ago) between *Paramecium tetraurelia* and *Tetrahymena thermophila* as a calibration point, we dated the emergence of *P. calkinsi* to approximately 611 million years ago—141 million years after the speciation of *P. bursaria*. Our analysis further revealed 195 expanded gene families and 2,800 contracted gene families in *P. calkinsi*. The expanded gene families were associated with oxidoreductase activity, ion binding, antiporter activity, transmembrane transport, and transport, likely reflecting the evolutionary adaptations to broad salinity ranges (Fig. 4E).

### Genome-wide analysis of mRNA alternative splicing

**Fig 5:**
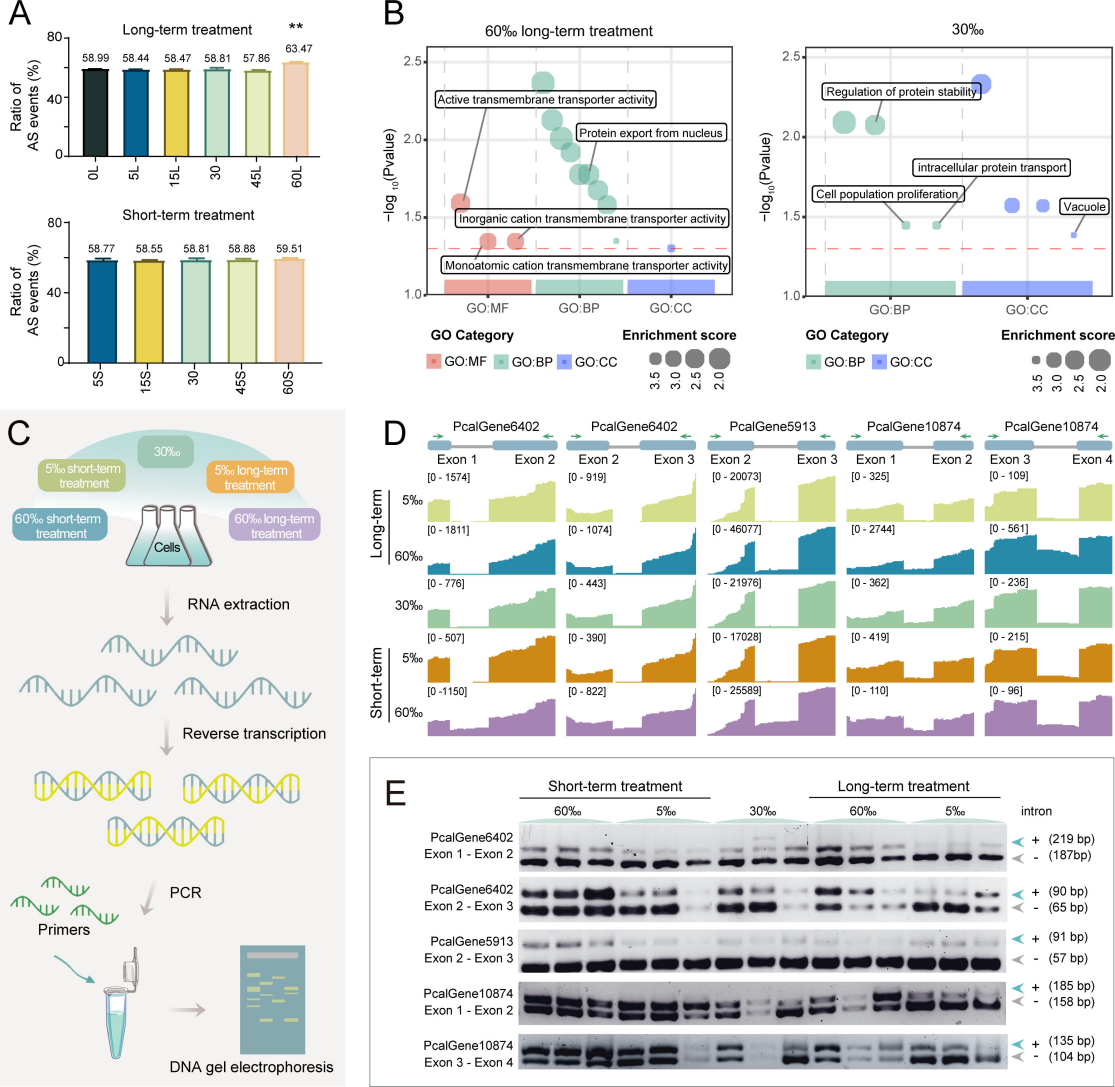
Alternative splicing frequency increases under osmotic pressures. (**A**) Alternative splicing (AS) ratio across cells under different osmotic pressures under long- (top) and short-term treatment (bottom). (**B**) Pathway enrichment analysis using the GO database for alternative splicing genes in 60‰ and 30‰ long-term treatment. (**C**) Schematic diagram of the experimental process for alternative splicing verification. (**D**) Intron structure and RNA-seq diagram of five representative genes. (**E**) DNA agarose gel electrophoresis of intron retention event verification under different osmotic pressure conditions. For each osmotic pressure treatment, the three gel lanes represent three independent biological replicates. The genes shown in panel **E** correspond directly to those presented in panel **D**. For each gene, the upper band represents transcript isoforms with a retained intron, and the lower band represents isoforms with the intron spliced out.

Because ATP-dependent activity and GTP binding, as well as RNA catalytic activity and ribonucleotide binding, were involved in cellular response to osmotic pressure changes, we then examined whether mRNA alternative splicing plays a role in response to these osmotic pressure changes. To detect structural variations of each transcript, we identified alternative splicing events using gffcompare with default parameters. In the 30‰ control sample, we detected alternative splicing in 58.81% of the genes (Fig. 5A). Under long-term salinity exposure, our results indicated that there was no obvious difference in hypoosmotic stress (58.99% in 0‰, 58.44% in 5‰, 58.47% in 15‰) and moderate hyperosmotic stress (57.86% in 45‰), whereas severe hyperosmotic conditions induced a substantial upregulation of alternative splicing events (63.47% in 60‰). Conversely, short-term treatments exhibited no significant alternative splicing alterations across all tested salinities (Fig. 5A).

A closer inspection revealed that intron retention frequency was significantly elevated in cells under long-term hyperosmotic stress at 60‰ (Fig. 5A). We performed a comparison of genes exhibiting unique alternative splicing events under long-term treatment conditions between 30‰ and 60‰ osmotic pressure treatments, followed by functional annotation analysis. The 30‰-specific alternatively spliced genes were primarily enriched in regulation of protein stability, cell population proliferation, and intracellular protein transport. In contrast, 60‰-specific alternatively spliced genes were predominantly associated with active transmembrane transporter activity, inorganic cation transmembrane transporter activity, and monoatomic cation transmembrane transporter activity (Fig. 5B). Though we acknowledge that this method, while identifying alternative splicing events, tends to overestimate their prevalence, these results supported that alternative splicing of specific genes played a role in regulating *Paramecium* gene expression and adaptation to hyperosmotic stress.

To validate the alternative splicing events under different osmotic pressure levels upon short- and long-term treatments, we reverse-transcribed mRNAs into cDNA and designed primers to amplify the sequences containing potentially retained introns using PCR (Fig. 5C, Table S7). The five introns in three genes highlighted in Fig. 5D were selected as representative examples based on their high expression levels, prominent intron retention, and consistent differential responses across salinity treatments. The retained introns showed two bands by agarose gel electrophoresis (Fig. 5E), which was in line with their transcriptome profiles by RNA-seq. Introns were more likely to be retained under the hyperosmotic pressure level (60‰). Through the annotation of gene function, these genes were found to play a role in signal transduction, material transport, and energy supply.

### Experimental validation of key differentially expressed genes

**Fig 6:**
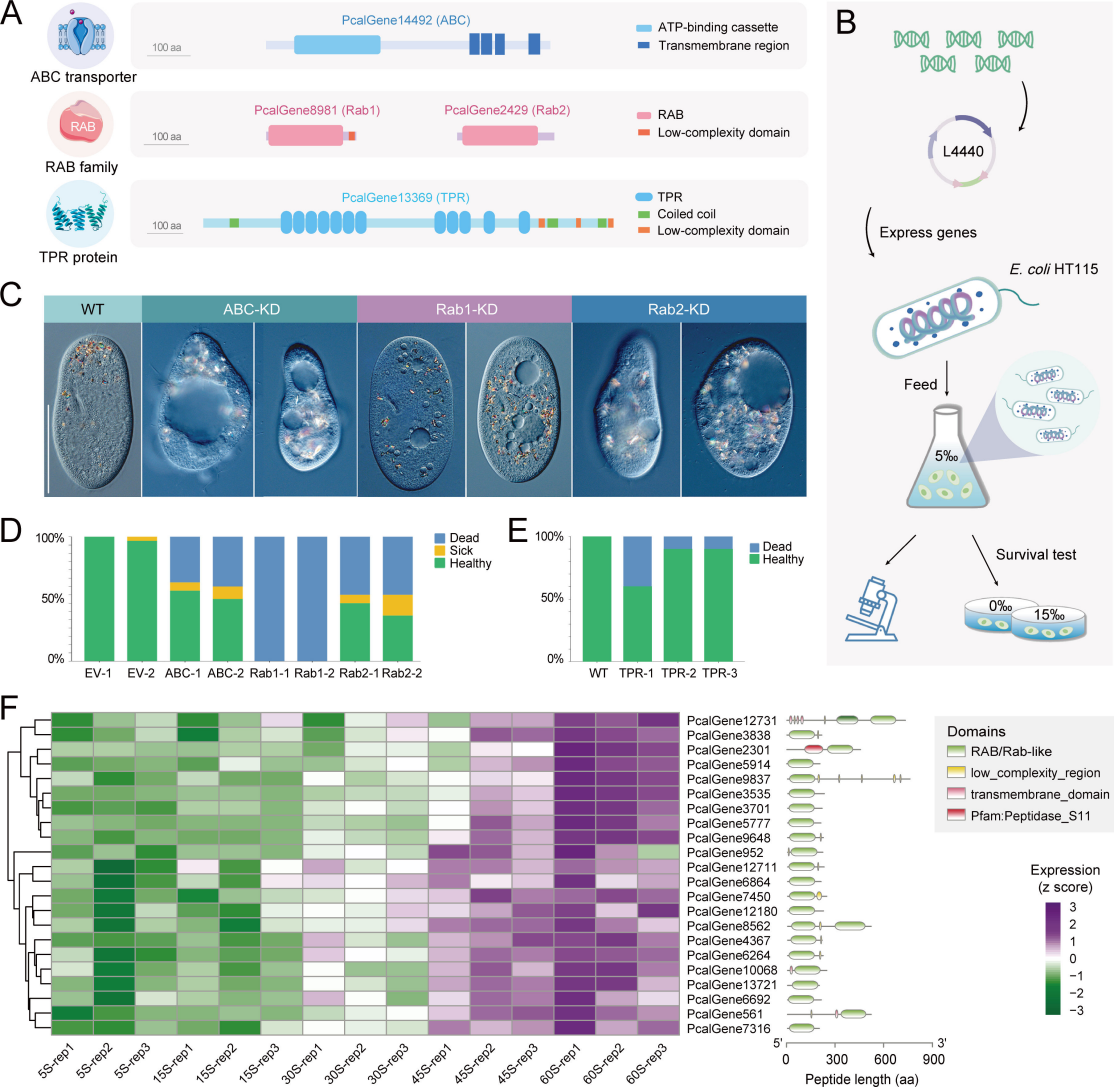
Experimental validation of key genes in response to osmotic pressure. (**A**) Predicted domains of four functional proteins. (**B**) Schematic diagram of the RNAi experimental process. (**C**) Photographs of *Paramecium calkinsi* control cells and after gene knockdown. Scale bar = 50 µm. (**D**) Statistical results of survival tests for cells transferred from 5‰ to 0‰ after gene knockdown. (**E**) Statistical results of survival tests for TPR-overexpressed cells transferred from 5‰ to 15‰. (**F**) The transcriptome profiles of Rab/Rab-like protein-coding genes with a gradient of differential expression upon short-term treatment. Conserved domains are denoted as rounded rectangles in different colors.

To experimentally validate the critical roles of the genes in osmoregulation, we selected three candidates. Their selection was based on two criteria: they were shared differentially expressed genes across multiple salinity conditions, and functional analyses strongly implicated them in osmotic regulation. The ATP-binding cassette (ABC) transporters were significantly enriched through the GO enrichment of the differentially expressed genes under different salinity treatments. The ABC transporter superfamily is a class of typical transmembrane transporters, with at least a core transmembrane domain (TMD) and a nucleotide-binding domain (NBD) (45, 46). They facilitate the translocation of a broad spectrum of substrates, such as ions, sugars, amino acids, polypeptides, toxic metabolites, and xenobiotics, across membranes by harnessing the energy released from ATP hydrolysis (47). We identified a potential ABC transporter protein (PcalGene14492) in *P. calkinsi*, with an NBD domain and a TMD domain (Fig. 6A). Compared to the control sample (30‰), it was significantly upregulated in the samples of 0‰ and 60‰. To verify the function of the ABC homolog, we performed RNAi-mediated knockdown by feeding *P. calkinsi* with *Escherichia coli* HT115 expressing dsRNA targeting the gene (Fig. 6B). Cells fed with HT115 bacteria containing empty L4440 plasmids were used as controls. Due to RNAi instability under high-salinity conditions, we conducted gene knockdown experiments in low-salinity medium (5‰). Compared to the normal morphology in the control cells (WT in Fig. 6C), RNAi knockdown (ABC-KD in Fig. 6C) caused a morphology defect, including contractile vacuole expansion and slow growth. After transferring the cells from 5‰ to 0‰ fresh water, there was no obvious impact for the control cells, while about half of the ABC transporter protein-depleted cells died (Fig. 6D). These results likely reflected the core role of the ABC transporters in the cellular response to the external osmotic stress.

Rab family of proteins represents the largest branch of the Ras-like small GTPase superfamily, which regulates the transmembrane traffic as a molecular switch by interacting with membrane bilayers and a variety of protein effectors (48). The Rab family comprises multiple functionally diverse proteins, which can coordinate with one another (49, 50). Previous research revealed that the number of Rab proteins may be linked to the flexibility of environmental adaptation (51, 52). In *T. thermophila*, 56 potential Rabs were identified, with TtRabD14 being localized to the contractile vacuole, an organelle that collects water from the cytoplasm and pumps it out of the cell to maintain osmotic balance (53). The large number of predicted Rab proteins indicates that some protozoa maintain a membrane compartment network at least as complex as that of multicellular organisms to adapt to changes in environmental conditions or life stages (53).

Compared with the Rab or Rab-like proteins in *T. thermophila*, over 87 putative proteins containing Rab or Rab-like domains were identified in *P. calkinsi* in the present study (Fig. S6). The abundance of Rab domain-containing proteins in *P. calkinsi* suggested a potential association of Rab proteins with osmotic pressure regulation. Remarkably, 22 *Rab-like* genes showed a dose-dependent upregulation in response to increasing osmotic pressure upon short-term treatments (Fig. 6F). These characteristics suggested that the Rab proteins may play a crucial role in osmoregulation. We identified two homologs of Rab protein in *P. calkinsi*, PcalGene8981 (Rab1) and PcalGene2429 (Rab2), which were then further validated through sensitive profile-based domain analysis (Fig. 6A). Similarly, we performed RNAi-mediated knockdown to verify the function of the Rab1/Rab2 homologs (Fig. 6B). After continuous feeding for more than seven days, compared to the normal state of control cells, knockdown of Rab1 resulted in the enlargement of the contractile vacuole, a slowdown of cell proliferation, and a gradual loss of viability, while Rab2 knockdown led to cell morphology defects and moderate vacuole enlargement (Fig. 6C). When transferred from 5‰ to fresh water (0‰), knockdown cells showed differential survival (Fig. 6D): no Rab1-silenced cells survived (0‰ viability), while only ~50% of Rab2-silenced cells remained viable.

We also tried to verify potential gene functions by overexpression through microinjecting the target genes with green fluorescent protein (GFP) tags into cells. PcalGene13369 was a tetratricopeptide repeat (TPR) protein, characterized by the presence of a TPR sequence motif (Fig. 6A). These repeats facilitate protein-protein interactions, allowing TPR proteins to function as scaffolds for the assembly of multiprotein complexes (54). A GFP tag was inserted after the start codon ATG of the *TPR* open reading frame, and the construct was microinjected into the macronucleus of *P. calkinsi* cultured under 5‰ salinity conditions. When transferred from 5‰ to 15‰ seawater, cells overexpressing the target genes showed varying degrees of viability loss, with 10%–40% viability reduction (Fig. 6E). It indicated that overexpression of *TPR* may impair the cell’s ability to respond to a higher osmotic pressure.

## DISCUSSION

### *Paramecium calkinsi* employs different molecular networks to cope with transient and long-term osmotic pressures

Osmotic pressure participates in various physiological activities of organisms, and isotonic osmotic pressure is a necessary condition for cells to exchange materials with their environment (55). For unicellular organisms, environmental changes in osmotic pressure directly influence intracellular osmotic balance, potentially disrupting tissue homeostasis and impairing cell development (21). Therefore, euryhaline unicellular organisms must possess remarkable adaptive mechanisms to survive drastic fluctuations in osmotic pressure (23). In the present study, we focused on the euryhaline unicellular eukaryote *P. calkinsi*, which exhibits an extraordinary capability to thrive across varying salinities. We explored the molecular basis underlying this adaptability by *de novo* assembling its macronuclear genome, conducting comparative genomic analyses with other freshwater species, analyzing differential gene expression under different osmotic pressures, and verifying some key genes by gene manipulation.

When cells are placed in a hypotonic environment, the influx of a large number of molecules may cause the cells to burst. To cope with this sudden pressure, cells have to rapidly dump intracellular solutes into the external medium. Aquaporin (Aqp1) is a key protein that helps freshwater ciliates adapt to hypotonic environments, typically showing high expression levels (56). However, in euryhaline *P. calkinsi*, the expression of the aquaporin homologous gene is relatively low and does not vary significantly across different salinity treatments. This suggests that *P. calkinsi* may employ different mechanisms to adapt to hypotonic conditions compared to freshwater ciliates, even within a shared environment.

In the present study, we found that the highly expressed genes under hypotonic pressures in short-term treatment are associated with extracellular organelle and trichocyst. This indicates that *P. calkinsi* may use these organelles to rapidly discharge intracellular solutes. While this response prevents lysis, it comes at the cost of diluting the cell’s internal contents, which can impair growth and metabolic functions. After the initial emergency response, cells must adjust their strategies to cope with sustained hypotonic stress.

In the long-term treatment under lower osmotic pressures, we found that ATPase-coupled transmembrane transporter activity, vesicle-mediated transport, oxidoreductase activity, cellular lipid metabolic process, and endomembrane system were significantly upregulated. Therefore, in a long-term hypotonic environment, *P. calkinsi* could avoid bursting by using ATPase transmembrane transport proteins to actively transport ions, thereby balancing osmotic pressure between the inside and outside of the cell. Additionally, it may regulate membrane tension through lipid metabolism and vesicle transport to adapt to osmotic fluctuations. Changes in osmotic pressure can also cause DNA damage and an increase in reactive oxygen species (ROS). Clearing ROS under salinity stress is crucial to prevent cell damage (57). The upregulation of oxidoreductase expression in the 0‰ long-term treatment group suggests that cells may be responding to the elevated ROS levels generated by osmotic stress (Fig. S3B).

On the contrary, when cells are placed in a hypertonic environment, the outflow of water from the cells will increase the solute concentration in the cell to an unsustainable level (58, 59). To counteract this situation, cells have to rapidly elevate the levels of osmolytes within the cytoplasm. For *P. calkinsi* under hypertonic pressures in long-term treatment, genes with enhanced transcription are related to amino acid biosynthesis, so that the cells can increase the synthesis of soluble substances. This critical adaptation restores the electrostatic balance and maintains cell functions; however, it imposes a substantial metabolic burden. To compensate, cells concurrently downregulate broader metabolic pathways, a strategy that optimizes survival by reallocating energy resources toward essential osmoregulatory processes.

In the 60‰ long-term treatment group, ATP-dependent activity, ABC-type transporter activity, ATPase-coupled transmembrane transporter activity, and primary active transmembrane transporter activity are significantly enriched. This suggests that in a hypertonic environment, cells may prevent shrinkage by accumulating soluble substances, thereby raising the intracellular solute concentration to counterbalance water loss. This process is highly energy-intensive, as it requires substantial ATP to synthesize these osmolytes and facilitate the transport of substances into the cell via transmembrane transport proteins. These adaptations could help ensure the stability of cellular osmotic pressure under sustained hypertonic stress.

In addition, most studies on osmotic regulation focus on the role of free amino acids and transaminases (35–37). In the long-term treatments, a subset of genes exhibited a pronounced upregulation specifically under the 45‰ salinity condition. These genes were functionally enriched in amino acid biosynthetic process, alpha-amino acid metabolic process, and small molecule biosynthetic process (Fig. 3C, cluster 6). This pattern likely reflects an adaptive strategy where organisms synthesize amino acids and other small molecules to maintain osmotic homeostasis during prolonged exposure to hyperosmotic stress. Notably, the expression levels of these genes declined at the extreme 60‰ salinity condition. Considering the observed poor physiological state of the organisms, this reduction may indicate a shift in cellular metabolism under critically high salinity. Concurrently, genes associated with cellular architecture maintenance (cytoskeletal protein binding, tubulin binding) were activated (Fig. 3C, cluster 1), suggesting the survival priorities shift from osmoregulation toward structural preservation as the primary cellular response to extreme osmotic stress.

### Cellular response to osmotic stresses includes oxidoreductase activity, transmembrane transport, and alternative splicing

Osmotic stress leads to water efflux or influx across the cell membrane, depending on the osmotic gradient, thereby disrupting osmotic homeostasis. Meanwhile, dysfunction of ion channels and transporters disrupts ion homeostasis, while osmotic stress activates cellular stress pathways, inducing excessive ROS production that damages membrane structures and biological macromolecules (60, 61). To cope with these stresses, cells must reprogram energy metabolism to meet the demands of ion transport and antioxidant responses, leading to increased metabolic burdens. The present analyses of gene family evolution revealed that *P. calkinsi* has shown significant expansion in gene families related to oxidoreductase activity, ion binding, and transmembrane transport. This appears to provide an evolutionary possibility (62) whereby adaptation to euryhaline environments is achieved through the expansion of these osmoregulatory genes.

Upon an osmotic shift, cells must rapidly adjust intracellular solute concentrations to maintain turgor pressure and avoid excessive cell swelling or shrinkage caused by osmotic imbalance. In order to cope with osmotic stress effectively, the primary response to this type of challenge must involve activation of existing transporters, since new enzyme systems would take too long to respond. There are two types of transport proteins that play a crucial role in the osmotic stress response: the classical ABC transporters, which are stimulated by changes in the internal ionic strength, and the BCCT transporters, whose activation is dependent on both a membrane trigger and a change in the cytoplasmic potassium (K^+^) concentration (63). In our research, both the enrichment of the differentially expressed genes and the ABC transporter depletion experiment demonstrated the significant role of ABC transporters in the osmotic response.

Rabs, predicted to act as determinants for complex membrane networks, are associated with organismal complexity (64). Research has shown that *Entamoeba histolytica* may possess up to 91 Rab genes, suggesting the potential to endow the cell with flexibility (65). Rab32 was shown to interact with V-H^+^-PPase in *Trypanosoma cruzi* to respond to osmotic stress (66). The multiple Rab proteins identified in *Tetrahymena* exhibit different subcellular localizations, with TtRabD14 and TtRabD2 located in contractile vacuole (53, 66), potentially suggesting that their function is associated with osmoregulation in ciliates. In our experiment, knockdown of Rab1 and Rab2 resulted in enlarged contractile vacuoles, showing their function in regulating the contractile vacuole. The numerous Rab domain-containing proteins identified in *P. calkinsi*, along with the potential trend of Rab gene expression to increase or decrease with changes in osmotic pressure, elucidate their crucial role in osmoregulation.

Alternative splicing is a crucial mechanism in gene expression that enables a gene to splice its precursor mRNA (pre-mRNA) in various ways, producing multiple distinct mature mRNA transcripts (67). These transcripts can encode proteins with diverse functions, thereby greatly expanding the genome’s coding potential and contributing to the remarkable diversity of the proteome (67, 68). In terms of adaptation and stress response, alternative splicing is essential for enhancing the cell’s ability to quickly react and adjust to environmental changes. Numerous studies have highlighted its pivotal role in regulating stress responses and facilitating cellular adaptation (69, 70). When comparing genes undergoing alternative splicing between the 60‰ long-term treatment group and the control group, we found that under hyperosmotic conditions, genes associated with active transmembrane transporter activity, inorganic cation transmembrane transporter activity, and monoatomic cation transmembrane transporter activity exhibited specific alternative splicing events. This suggests that alternative splicing could represent a regulatory strategy enabling organisms to cope with hyperosmotic stress.

### Conclusion

**Fig 7:**
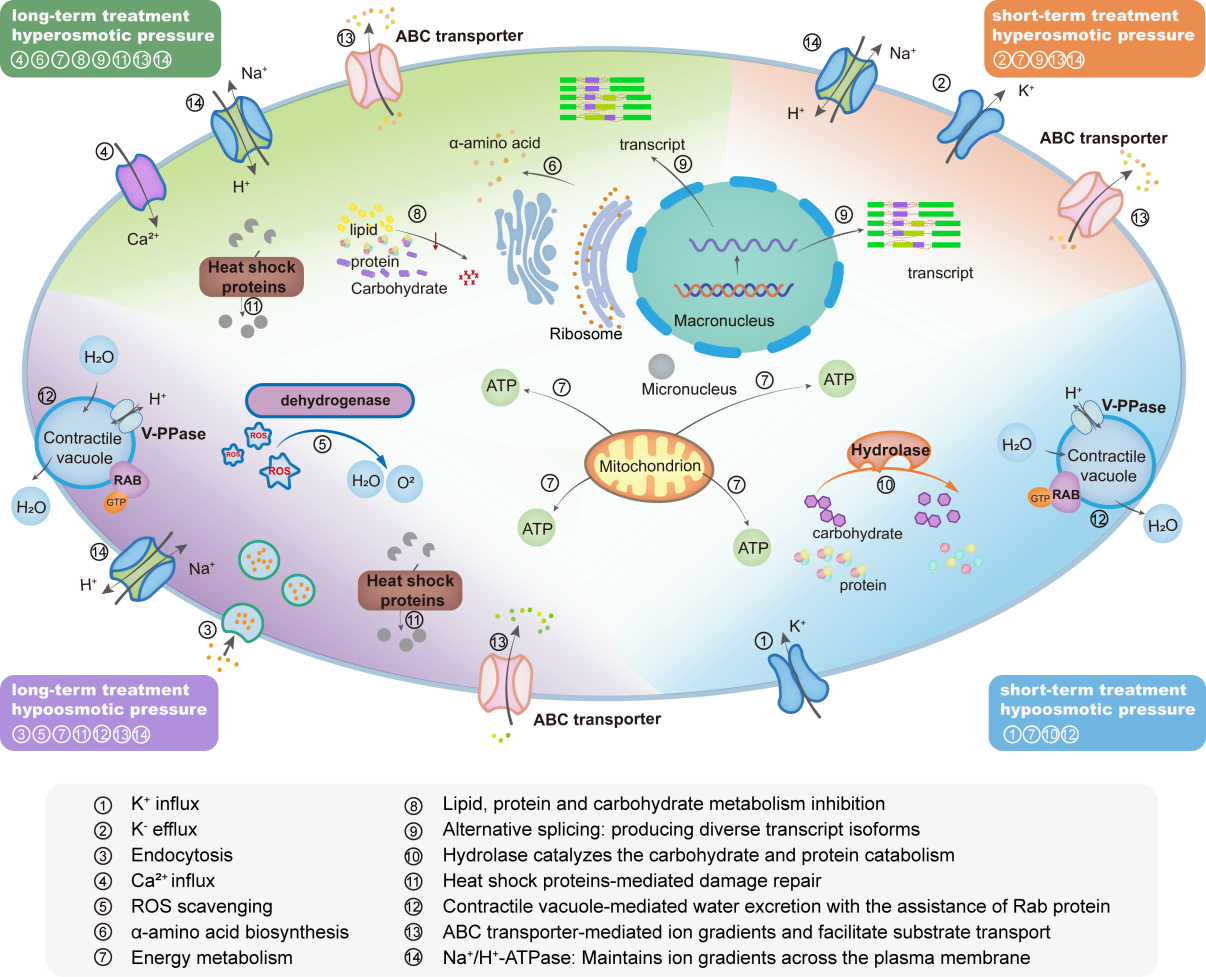
Schematic model of biological pathways in *Paramecium calkinsi* under different osmotic pressures Different colored sections represent specific treatments: long-term treatment under hyperosmotic pressure (green), long-term treatment under hypoosmotic pressure (purple), short-term treatment under hyperosmotic pressure (orange), and short-term treatment under hypoosmotic pressure (blue). Cellular components and processes are labeled with numbers, corresponding to the pathways listed at the bottom. Proteins playing critical roles in these pathways are highlighted in bold.

Euryhaline unicellular organisms must possess remarkable adaptive mechanisms to survive drastic fluctuations in osmotic pressure. In this study, we investigated the molecular basis underlying the osmoregulation capability in the single-celled euryhaline ciliate *P. calkinsi* through a combination of bioinformatic analyses and molecular experiments. We obtained the high-quality assembly of its somatic macronuclear genome for the first time. To understand how *P. calkinsi* manages osmotic stress, we explored its molecular basis governing short-term versus long-term adaptation across varying osmotic pressure levels. Differential gene expression analysis revealed regulatory pathways critical for osmoregulation and genes that exhibit osmosensitivity. Cells need to coordinate multiple physiological processes to cope with osmotic pressure stress. Key processes include the regulation of transmembrane transport proteins, cytoskeletal rearrangements, and energy-dependent pathways. Ion channels and ATPase enzymes may play pivotal roles in maintaining osmotic balance by controlling the influx and efflux of ions and water, while cytoskeletal dynamics are crucial for maintaining cell integrity under stress. Additionally, the upregulation of stress-responsive proteins, such as heat shock proteins and osmolyte-producing enzymes, provides further resilience. Gene family expansions and mRNA alternative splicing related to ion binding, cytoskeletal activity, and ATP-dependent processes likely underpin *P. calkinsi*’s broad osmotic adaptability. Moreover, cellular energy supply also plays a crucial role in these processes. Here, we categorize biological pathways and their roles under different osmotic pressures, proposing a model to demonstrate *P. calkinsi*’s molecular strategies for osmotic stress tolerance (Fig. 7). In summary, this study provides insights into the molecular basis of how free-living single-celled eukaryotes adapt to extreme osmotic pressures across both short- and long-term stress timescales from both evolutionary and experimental biology standpoints. Though a substantial effort will be required to systematically validate the function of each candidate gene and elucidate their associated pathways, this work constitutes a valuable foundation for future function-driven research.

## MATERIALS AND METHODS

### Cell culture and sample preparation

The strain of *P. calkinsi* used in this study was collected from a brackish water area with a salinity of 25‰ near an estuary in Yantai, China (Fig. 1A). Cells were cultured daily in filtered and sterilized natural seawater with a salinity of 30‰ in the laboratory. *Escherichia coli* was provided as the food source.

Salinity gradients were adjusted by diluting a self-prepared 100‰ salinity salt solution, consisting of NaCl (~1.60 M), MgCl_2_ (~81.90 mM), KCl (~35.73 mM), CaCl_2_ (~27.03 mM). For long-term treatments, monoclonal cell lines were maintained at salinities of 0‰, 5‰, 15‰, 30‰, 45‰, and 60‰ for at least 1 month. For each treatment, three biological replicates were performed, and the detailed operations were as follows: briefly, a single cell was isolated and cultured to establish a monoclonal cell line, which was transferred from a salinity of 30‰ to the target salinity medium for at least 2 weeks in approximately 0.5 mL of medium, during which only a small number of divisions occurred. Then, cells from this pre-adapted monoclonal line were isolated and cultured in the corresponding salinity medium to a volume of 30 mL (with an approximate cell density of 2,300 cells/mL and a division cycle of ~8 h per generation in 27°C). This process took about 5–6 days, during which cells divided for 15–24 generations. Afterward, the cells were maintained in the corresponding salinity medium for about 1 week with very limited divisions. Subsequently, the culture was expanded to 400 mL for the following RNA extraction (0‰, 5‰, 15‰, 30‰, 45‰, and 60‰) or DNA extraction (5‰, 30‰, and 45‰), which requires 2–3 days for divisions and food consumption.

For short-term experiments, cells were initially cultured in 30‰ salinity and then subjected to various salinity conditions (0‰, 5‰, 15‰, 30‰, 45‰, and 60‰) for 1 h before RNA extraction (Fig. 2A). Similarly, three biological replicates were performed for each treatment. Notably, the cells transferred to 0‰ were too fragile to collect. Therefore, the transcriptome of the short-term treatment under 0‰ salinity was missing.

Cells were concentrated by centrifugation at 1,000 g for 3 min. Genomic DNA was extracted using the MagAttract HMW DNA Kit (QIAGEN, Germany, Cat. No. 67563) according to the manufacturer’s instructions. Total RNA was extracted from cells exposed to both long-term and short-term treatments using the RNeasy Kit (QIAGEN, Germany, Cat. No. 74106) (Fig. 2A).

### High-throughput sequencing

The isolated DNA was sequenced using both PacBio CLR sequencing and short-read PE150 sequencing (Fig. 1B; Fig. S1). The Single Molecule Real-Time Sequencing genomic DNA libraries for CLR sequencing were constructed with an average insert size of 30 kb following the manufacturer’s recommended protocols and sequenced on the PacBio Sequel II platform (BGI, Qingdao, China). For short-read PE150 sequencing, short-insert-size (200 bp–400 bp) genomic DNA libraries were constructed and then subjected to the DNBSEQ-T1&T5 sequencing platform (BGI, Qingdao, China). For mRNA sequencing, polyA-tailed transcripts were enriched using Oligo(dT) magnetic beads, fragmented, and reverse transcribed into double-stranded cDNA using N6 primers. Adapters were ligated to the cDNA fragments, and a single-stranded circular DNA library was prepared. The library was sequenced on the BGISEQ-500RS platform, generating paired-end 100 bp reads (BGI, Qingdao, China).

### Genome assembly

Approximately 3,240× PacBio continuous long reads were split into groups, each containing 180× reads, and reads in each group were respectively corrected using Canu v.2.2 (71). The corrected reads were assembled using Canu v.2.2 (71) with cleaning and duplication purged. On the other hand, the corrected reads were split into groups with 40× reads for each and reads in each group were assembled using Smartdenovo v.1.0.0 (72), respectively. The assemblies generated from Canu and Smartdenovo were merged using Quickmerge (73). Duplications were purged from the merged assembly to generate the haploid assembly. The final assembly was polished by PacBio CLR data and short reads using Nextpolish v.1.4.1 (74). Mitochondrial DNA sequences of ciliates and bacterial genomic sequences were downloaded from GenBank as the alignment database, and contamination caused by mitochondria or bacteria was identified using BLAST (BLAST E-value cutoff = 1e − 5) and then removed. The motif of telomeres was identified using MEME (75) and then detected and counted using a custom Perl script that recognized the telomere repeat 6-mer 5′-(C_4_A_2_)_n_-3′ at the ends of scaffolds. Genome assembly was evaluated using Quast v.5 (76) and BUSCO v.5.2.1 (77).

### Genome annotation

The genome annotation was performed on the final assembly (Fig. 1B; Fig. S1). *De novo* prediction of genes (Gene model 1) was performed using Augustus v.3.5 (78). RNA-seq reads were mapped to the genome assembly using HISAT2 v.2.1.0 (79). Mapping results for RNA-seq reads were visualized using IGV (80). Based on the RNA-seq reads mapping results, genome annotation (Gene model 2) was further performed using StringTie v.1.3.3b (81). The predicted transcripts by StringTie were further aligned to the genome assembly using Gmap v.2017-09-15 (82) and were used to calculate the WAM model (Gene model 3) using EuGene v.4.2a (83). These three gene models were combined to generate the final gene model, optimized by the proteome data (84) of three model ciliates (*Paramecium tetraurelia*, *Paramecium caudatum*, and *Tetrahymena thermophila*). The sequence motif of intron regions was identified using MEME (https://meme-suite.org/meme). Repeats in the genome assembly were annotated by combining *de novo* prediction and homology searches using RepeatMasker (-engine rmblast -species ‘paramecium’ -q) (85). The tRNA and other ncRNA genes were detected in the macronuclear genome by tRNAscan-SE v.1.3.1 and Rfam v.11.0, respectively (86, 87). Predicted protein products were annotated by alignment to domains in the Pfam-A database using InterProScan v.5.56 and to protein sequences of other *Paramecium* species from ParameciumDB (https://paramecium.i2bc.paris-saclay.fr) using BLAST+ v.2.3.0 (E-value cutoff = 1e − 5) (88, 89). Genome assemblies of other *Paramecium* species were acquired from ParameciumDB (https://paramecium.i2bc.paris-saclay.fr). The frequency of stop codon usage was estimated by a custom Perl script that recognized the potential stop codons at the end of coding sequence regions that share strong homology with close relatives of other *Paramecium* species. Whole-genome duplication analysis was performed using MCScanX and ParaAT based on the cDNA sequences of the annotated genes (90, 91).

### Differential gene expression analysis

Reads mapped to each gene locus were quantified using featureCounts (92). Differential gene expression analysis and principal component analysis were performed using the R package DESeq2 (|log2 fold-change [log2FC]| > 1, adjusted *P*-value <0.1) (93). Weighted gene co-expression eigengene network analysis was performed using WGCNA (SoftPower = 20) (94). GO term enrichment analysis was carried out using g: Profiler (https://biit.cs.ut.ee/gprofiler/gost). The bubble plot was generated by the R package ggplot2 (95, 96). To cluster the genes identified in the different treatments, we used the R package Mfuzz (http://www.bioconductor.org/) (97), which performs fuzzy c-means clustering (c = 6, m = 1.8).

### Alternative splicing analysis

StringTie v.2.2.1 (81) was used to assemble transcripts independently for each sample, guided by the reference genome. The resulting individual transcriptome files (in GTF format) were then merged using the StringTie --merge command to generate a comprehensive, non-redundant set of transcript models. This merged transcriptome was compared to the reference annotation using gffcompare v.0.12.6 (default thresholds, -e 100 -d 100) to classify the novel and known transcripts (81). Finally, alternative splicing events were categorized according to transcript classification codes (https://ccb.jhu.edu/software/stringtie/gffcompare.shtml) and quantified from the merged GTF file in R. Statistical significance between different groups was analyzed using the *t*-test, with significance levels denoted as follows: *P* < 0.05 (*) and *P* < 0.01 (**). Intron retention evaluation analysis was performed using IRFinder v.1.3.1 (parameters: length of non-overlap part of an intron > 10 nt and more than half of this intron), and intron retention events that occurred in at least one-fifth of all samples were used for further analysis (98).

### Copy number variation and structural variant calling

Paired-end reads from short-read sequencing of genomic DNA were mapped to the genome assembly using BWA-MEM (99). Copy number variation was calculated based on genomic coverage using function multiBigwigSummary of deeptools v.3.5.3 (100). Synteny groups were identified from the conserved linkages between the chromosomes using BLAST+ v.2.3.0 (E-value cutoff = 1e − 200, identity cutoff = 99, match length cutoff = 1,000) (88). Structural variant calling was performed using Dysgu (101). The copy number variation, structural variants, and synteny groups were visualized using the R package Rcircos (102).

### Phylogenetic analysis and gene family expansion analysis

Orthologous protein sequences were identified among *P. calkinsi* (the current work), 15 *Paramecium* species (including 21 strains, ParameciumDB: https://paramecium.i2bc.paris-saclay.fr), and *T. thermophila* (as an outgroup, TGD: https://tet.ciliate.org) for phylogenomic analysis using OrthoFinder v.2.5.4 (-S diamond -M msa -T raxml -I 1.5) (103). The ultrametric phylogenetic tree was based on a rooted species tree inferred by OrthoFinder, with divergence time estimation performed using the mcmctree module implemented in PAML v.4.10.9 (104). The rates of amino acid substitution were estimated using the Codeml program implemented in the PAML software package (104). Convergence of the inferred phylogenetic trees was subsequently validated using Tracer v.1.7.2 (105). Trees were visualized using MEGA v.7.0.20 (106). Significantly expanded or contracted gene families were identified by CAFE (Computational Analysis of [gene] Family Evolution) v.5.0 (-k 5) (107). The divergence time between *Paramecium tetraurelia* and *T. thermophila* (662–934 Mya) was adopted as the calibration point (108). Functional enrichment analysis of expanded and contracted gene families was performed using TBtools-II (109).

### Gene overexpression

The open-reading frames of gene *TPR,* as well as putative regulatory regions, were amplified by PCR and cloned into the pCE2 TA/Blunt-Zero vector using 5 min TA/Blunt-Zero Cloning Kit (Vazyme, China, Cat. No. C601-02). The codon-optimized GFP (723 bp, accession number AB071703 in GenBank [110]) was inserted after the start codon ATG by ligation of ends generated by the *BsaI* restriction enzyme (NEB, USA, Cat. No. R3733V). The recombinant plasmid was transformed into *E. coli* DH5α and extracted using the QIAGEN PlasmidPlus Midi Kit (QIAGEN, Germany, Cat. No. 12943). After digestion by the restriction enzyme *AhdI* (NEB, USA, Cat. No. R0584S), the linear DNA was injected into the macronucleus of the cell by microinjection (111). GFP-specific primers were used to detect whether the fusion gene was kept in the cell (Table S7).

### Alternative splicing verification

Total RNA was extracted from cells under different salinity treatment conditions using Trizol, and cDNA was synthesized via the reverse transcription reagent Hifair AdvanceFast 1st Strand cDNA Synthesis Kit (YEASEN, China, Cat. No. 11149ES10) with Oligo d(T)_18_ primers according to the manufacturer’s instructions. The reverse transcription product was then used as a template for PCR amplification to verify alternative splicing, using PrimeStar Max Premix (TaKaRa, Japan, Cat. No. AMF1073A) and Taq master mix (Vazyme, China, Cat. No. 7E790B3). The primers used for alternative splicing verification are listed in Table S7.

### RNA interference

Genomic DNA was provided as a template for PCR for the target genes. The PCR products were subsequently ligated into the pCE2 TA/Blunt-Zero vector using a 5 min TA/Blunt-Zero Cloning Kit (Vazyme, China, Cat. No. C601-02), and then transferred to *E. coli* DH5α receptor cells. Positive clones were identified and selected for plasmid extraction. A double restriction-enzyme digestion segment generated using *XhoI* (NEB, USA, Cat. No. R0146L) and *SacII* (NEB, USA, Cat. No. R0157L) from a pCE2 plasmid was ligated into the L4440 plasmid, which contained two T7 promoters. Subsequently, the recombinant plasmid was transferred to HT115 competent cells, and positive clones were spread on a plate (LB medium containing tetracycline and ampicillin) for storage. The positive clone was inoculated into LB (with ampicillin) culture medium and shaken overnight at 37°C, then the OD value was detected and diluted to 0.07 with 5% fresh lettuce juice (diluted with modified Dryl solution using KH_2_PO_4_ instead of NaH_2_PO_4_ [KDS]) (112–114), and IPTG (2 mmol/L) was added to induce dsRNA expression for 6 h. The medium containing the induced bacteria was then used to culture the cells. HT115 bacteria containing empty L4440 plasmids were used as a control.

## Data Availability

The Illumina and PacBio sequencing data of the MAC genome, along with the Illumina transcriptome data, have been deposited in GenBank under BioProjects PRJNA1224858 and PRJNA1223004, respectively. The accession number for the genome assembly is JBTISH000000000.

## SUPPLEMENTAL MATERIAL

Supplemental material mbio.03032-25-s0001.docx Figure S1-S6 and supplemental table list.

Table S1 mbio.03032-25-s0002.xlsx Information of somatic genome assemblies of *Paramecium* species.

Table S2 mbio.03032-25-s0003.xlsx Classification of RNA types, subtypes, and quantity statistics.

Table S3 mbio.03032-25-s0004.xlsx GO pathway enrichment analysis results of the DEGs under different osmotic conditions.

Table S4 mbio.03032-25-s0005.xlsx GO pathway enrichment analysis results of WGCNA gene modules with increased expression under hyperosmotic or hypoosmotic pressures upon long-term treatment.

Table S5 mbio.03032-25-s0006.xls CNVs and gene expression correlations under 5‰ and 45‰ osmotic pressures.

Table S6 mbio.03032-25-s0007.xls MCMC convergence diagnostics for Bayesian divergence time estimation.

Table S7 mbio.03032-25-s0008.xlsx Information on relevant genes and the primers used.

ASM does not own the copyrights to Supplemental Material that may be linked to, or accessed through, an article. The authors have granted ASM a non-exclusive, world-wide license to publish the Supplemental Material files. Please contact the corresponding author directly for reuse.
