# Supplementary material for: Molecular basis of the short- and long-term osmoregulation capability in the euryhaline unicellular eukaryote Paramecium calkinsi
Source: mBio. 2026 Mar 6;17(4):e03032-25. doi: 10.1128/mbio.03032-25 (PMC13059789; doi:10.1128/mbio.03032-25)
Supplement: Supplemental material — Figure S1-S6 and supplemental table list. [file mbio.03032-25-s0001.docx]

**Supplementary materials**

**Molecular basis of the short- and long-term osmoregulation capability in the euryhaline unicellular eukaryote *Paramecium calkinsi***

Jia Liu^1, ‡^, Juan Yang^2, ‡^, Xue Zhang^1, ‡^, Rui Wang^1^, Lei Yang^2^, Huan Dou^2^, Rebecca A. Zufall^3^, Xiao Chen^2, *^, Feng Gao^1,4, *^

^1^ Key Laboratory of Evolution & Marine Biodiversity (Ministry of Education), and Institute of Evolution & Marine Biodiversity, Ocean University of China, Qingdao 266003, China.

^2^ Marine College, Shandong University, Weihai 264209, China.

^3^ Department of Biology and Biochemistry, University of Houston, Houston TX, USA 77204

^4^ Laboratory for Marine Biology and Biotechnology, Qingdao Marine Science and Technology Center, Qingdao 266237, China.

*Corresponding authors: Feng Gao, Key Laboratory of Evolution and Marine Biodiversity (Ministry of Education), Institute of Evolution and Marine Biodiversity, Ocean University of China, 5 Yushan Road, Qingdao, Shandong 266003, China. Email: [gaof@ouc.edu.cn](mailto:gaof@ouc.edu.cn); Xiao Chen, Marine College, Shandong University, Weihai 264209, China. Email: xc@sdu.edu.cn.

^‡^ Jia Liu, Juan Yang and Xue Zhang contributed equally to this work.


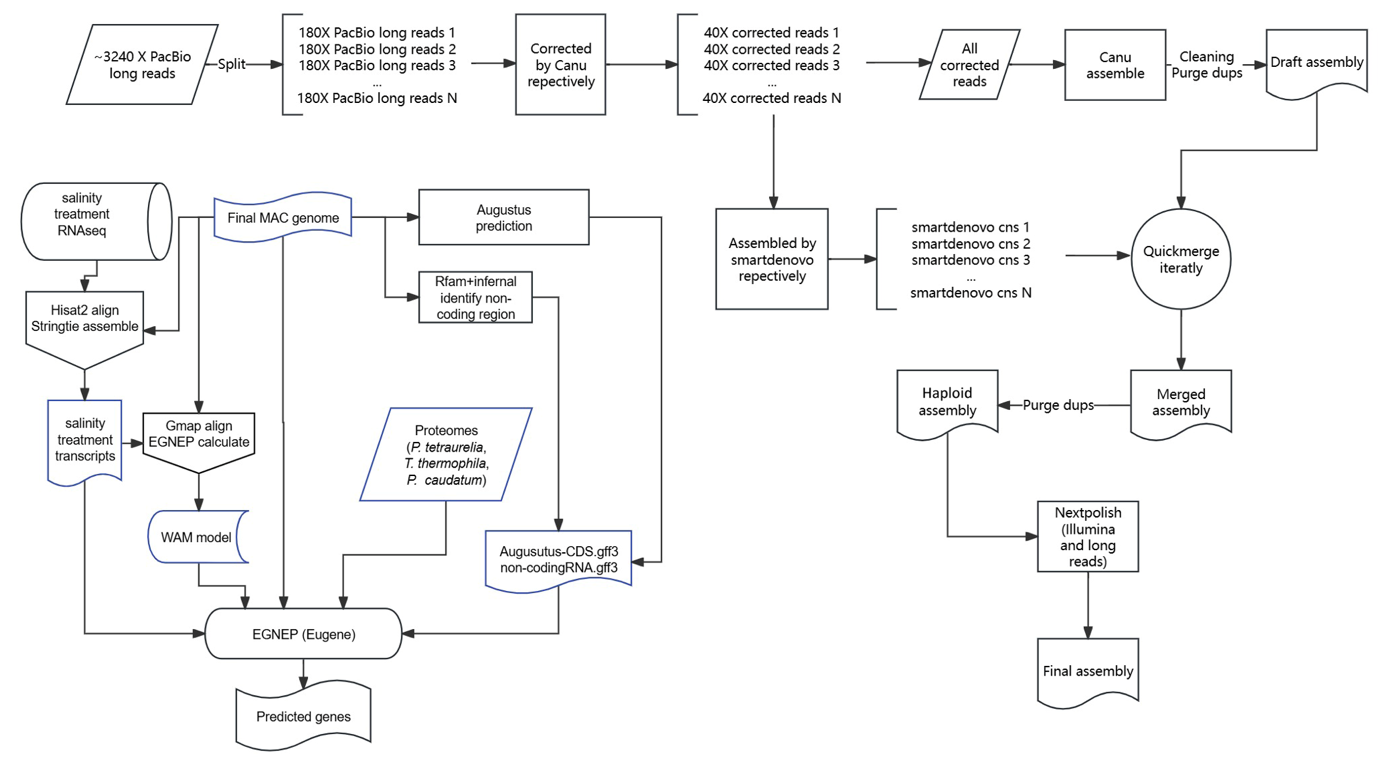


**Figure S1. Schematic diagram of the strategy for the genome assembly and annotation, related to Figure 1.**

The primary genome assembly of *P. calkinsi* was conducted using two assembly software

programs, Canu and Smartdenovo, along with an iterative merging strategy. The assembly was then polished by high-depth sequencing data, and redundancy was eliminated to obtain the haploid genome. Eugene was used to predict gene models in P. calkinsi genome, integrating evidence from multiple resources.

**
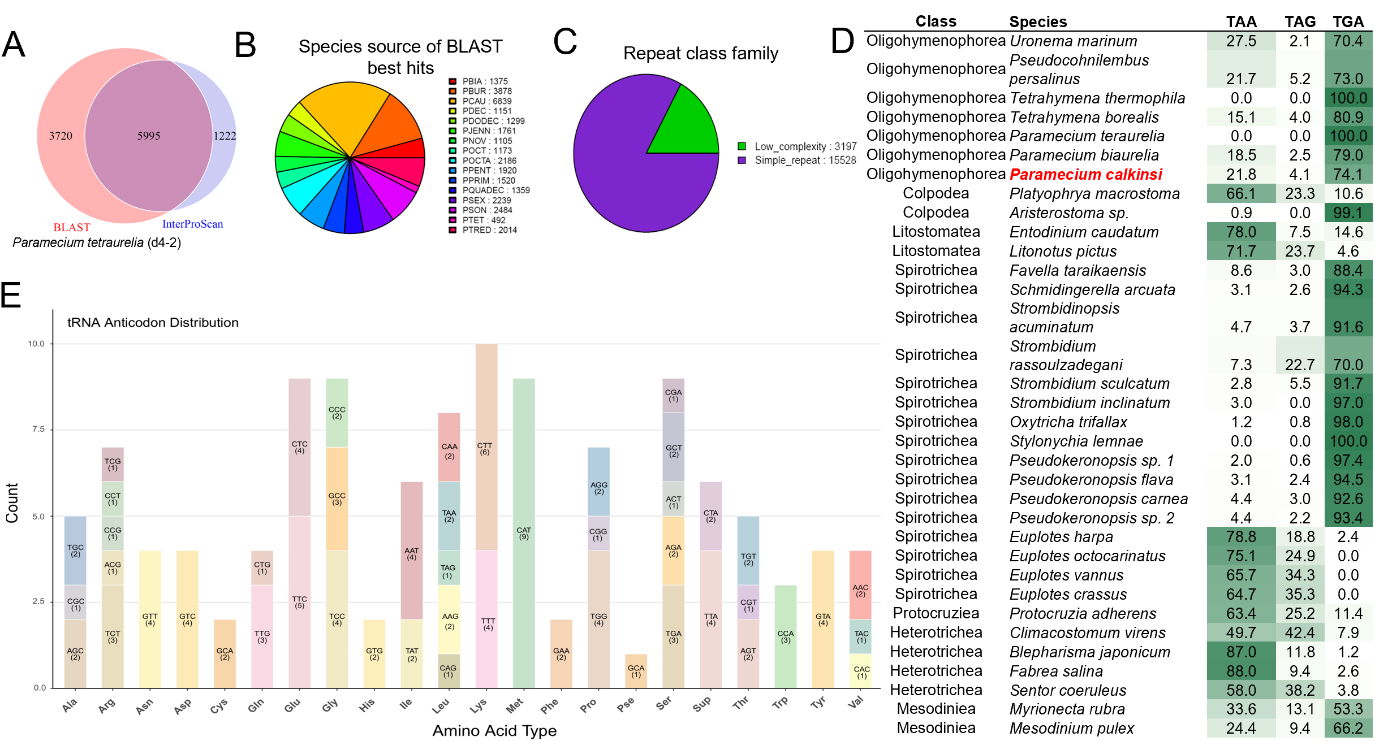
**

**Figure S2. Genomic features, related to Figure 1.**

(A) Venn diagram showing the overlap between gene annotation results using BLAST and InterProScan. (B) Pie chart showing species source of BLAST best hits of *P. calkinsi* protein sequences in ciliate protein database. (C) Pie chart showing the annotation results of repeat sequences in *P. calkinsi* genome. (D) Summary of stop codon usage information of ciliates including *P. calkinsi*. Species from the current work is denoted in red. (E) Distribution of tRNA anticodon. Different colors represent specific anticodons and their counts.


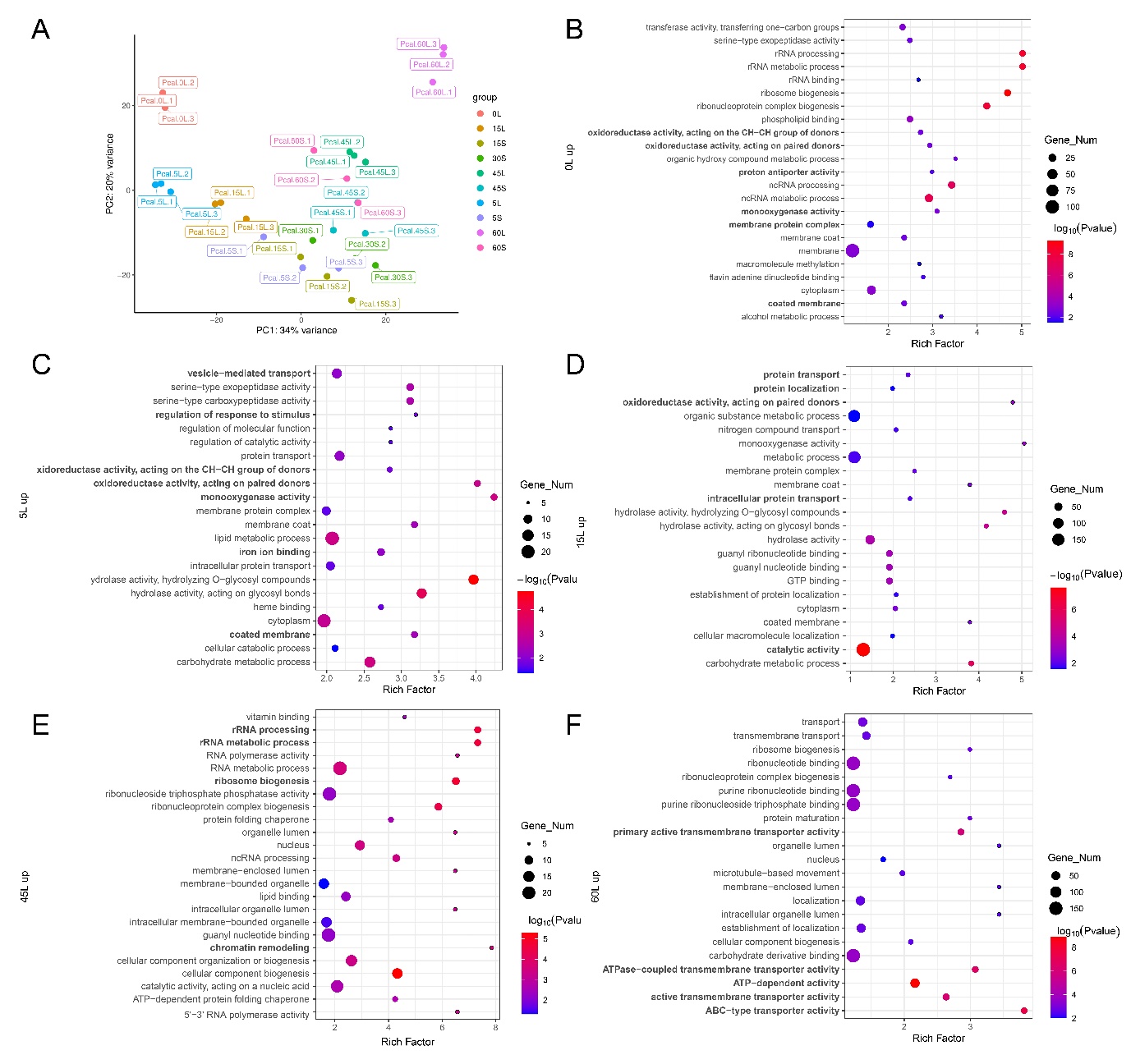


**Figure S3. Gene expression changes under osmotic pressures, related to Figure 2.**

(A) PCA analysis for cell samples under hypoosmotic (0‰, 5‰, 15‰), normal osmotic pressure (30‰) and hyperosmotic pressures (45‰, 60‰) upon long- (L) and short-term treatment (S). (B-F) GO enrichment analysis of the up-regulated genes in 0‰ salinity long-term treatment (B), 5‰ salinity long-term treatment (C), 15‰ salinity long-term treatment (D), 45‰ salinity long-term treatment (E), 60‰ salinity long-term treatment (F).


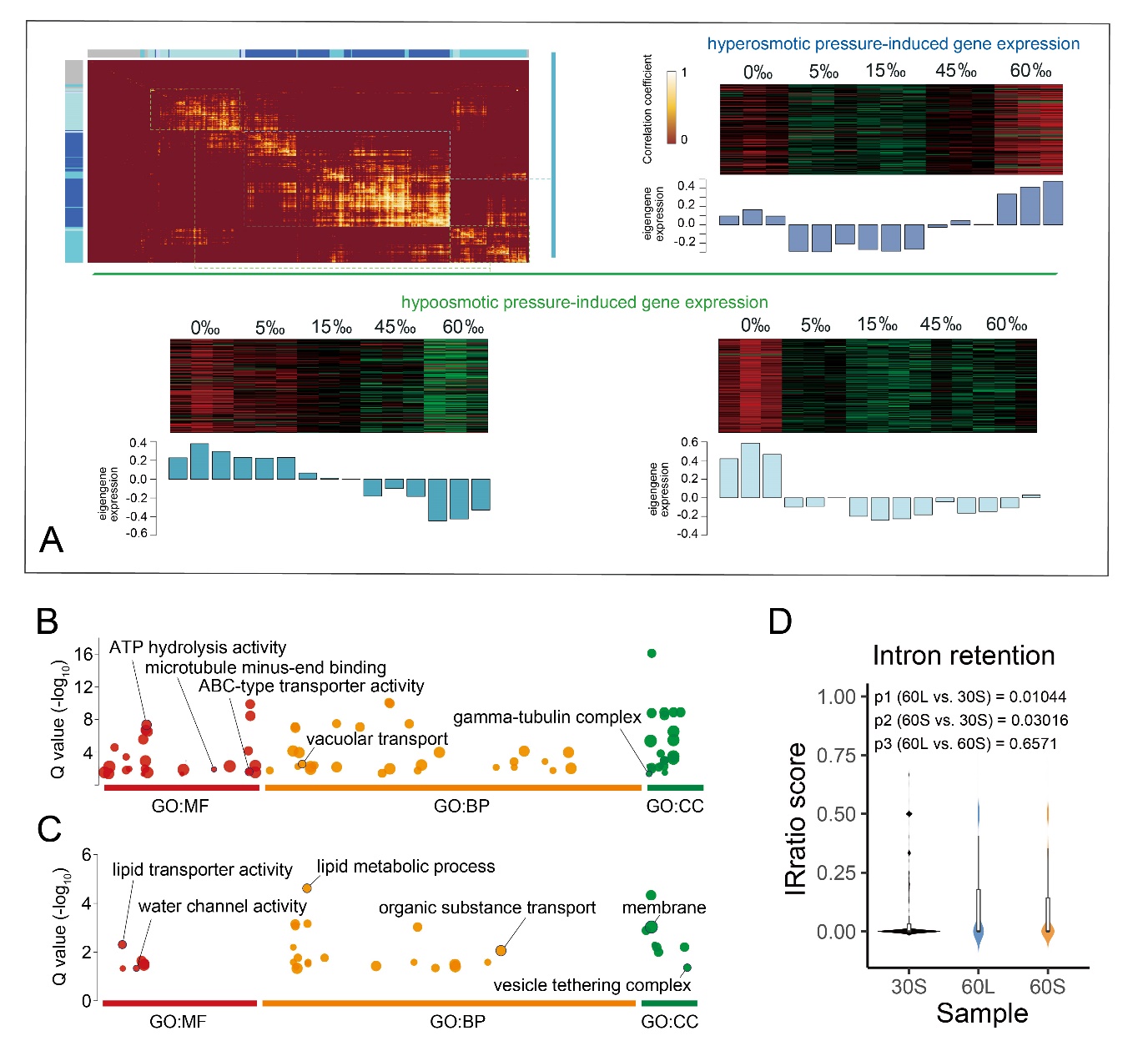


**Figure S4. Differentially expressed genes shared by osmotic pressures, related to Figure 3.**

(A) Weighted gene co-expression network analysis (WGCNA) for cells cultured under different osmotic pressures upon long-term treatment. Two largest gene co-expression modules under hyperosmotic and hypoosmotic pressure are highlighted in blue and green, respectively. (B-C) Pathway enrichment analysis for the co-expression modules under hyperosmotic (blue), corresponding to modules in Figure S4A. (C) Pathway enrichment analysis for the co-expression modules under hypoosmotic pressure (green), corresponding to modules in Figure S4A. (D) Intron retention evaluation analysis for cells treated upon hyperosmotic pressure level (60‰) for either long- or short-term treatment, using 30‰ as control.


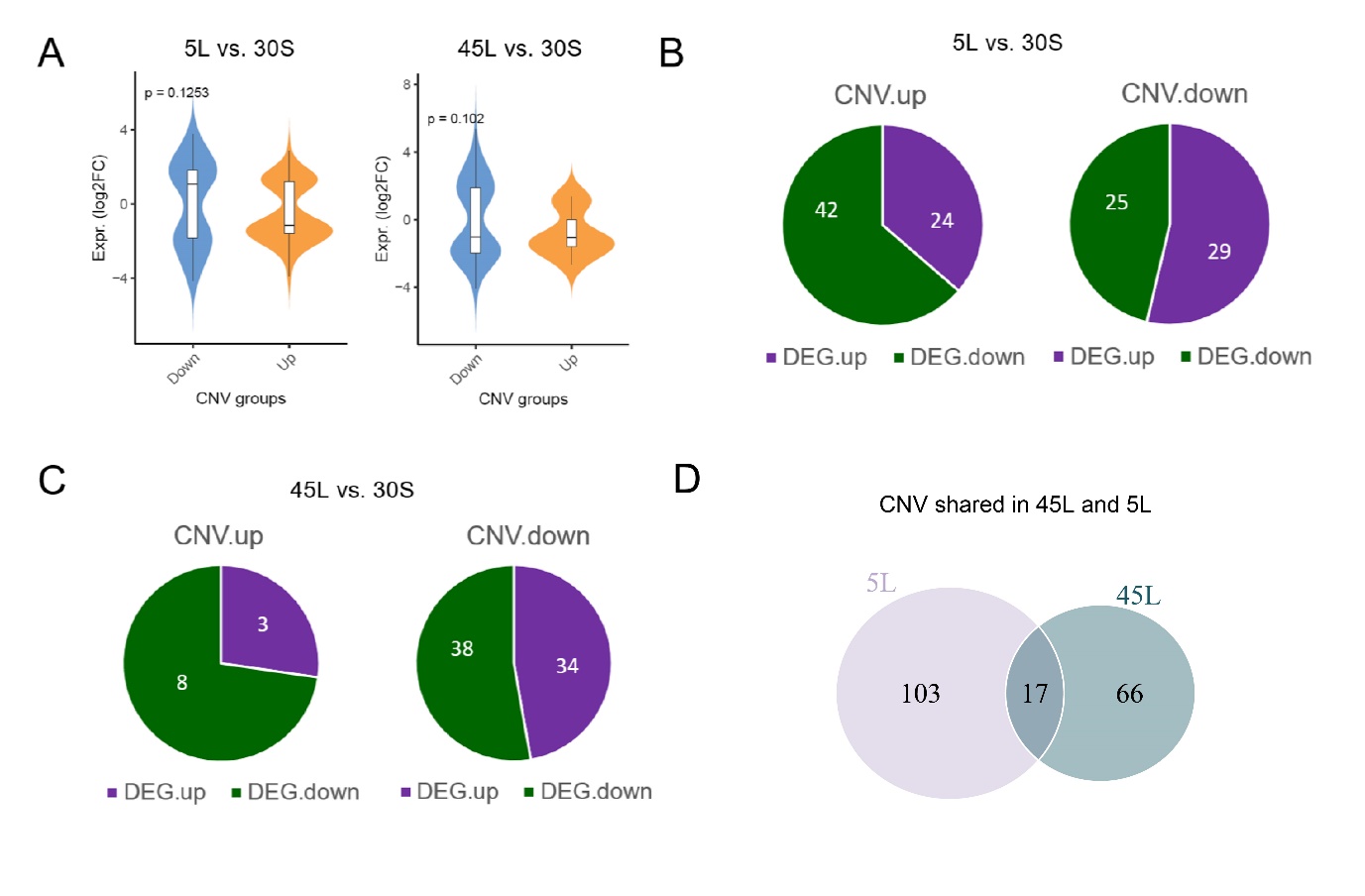


**Figure S5. Genes with copy number variations upon osmotic pressures are not differentially expressed, related to Figure 4.**

(A) Copy number variations (CNVs) in up- and down-regulated genes in the cells upon either the long-term hypoosmotic pressure (5‰, left) or the long-term hyperosmotic pressure (45‰, right), comparing to that of the control condition of short-term normal osmotic pressure 30‰. (B) Gene expression changes in genes with increased and decreased copy number in the cells upon either the long-term hypoosmotic pressure (5‰, left) or the long-term hyperosmotic pressure (45‰, right), comparing to that of the control condition of short-term normal osmotic pressure 30‰. (C) Pie diagram showing the numbers of up- and down-regulated genes among the genes with increased (left) and decreased (right) copy number in the cells upon the long-term hyperosmotic pressure (45‰). (D) Venn diagram of copy number variations in 5‰ and 45‰ salinity long-term treatment.

**
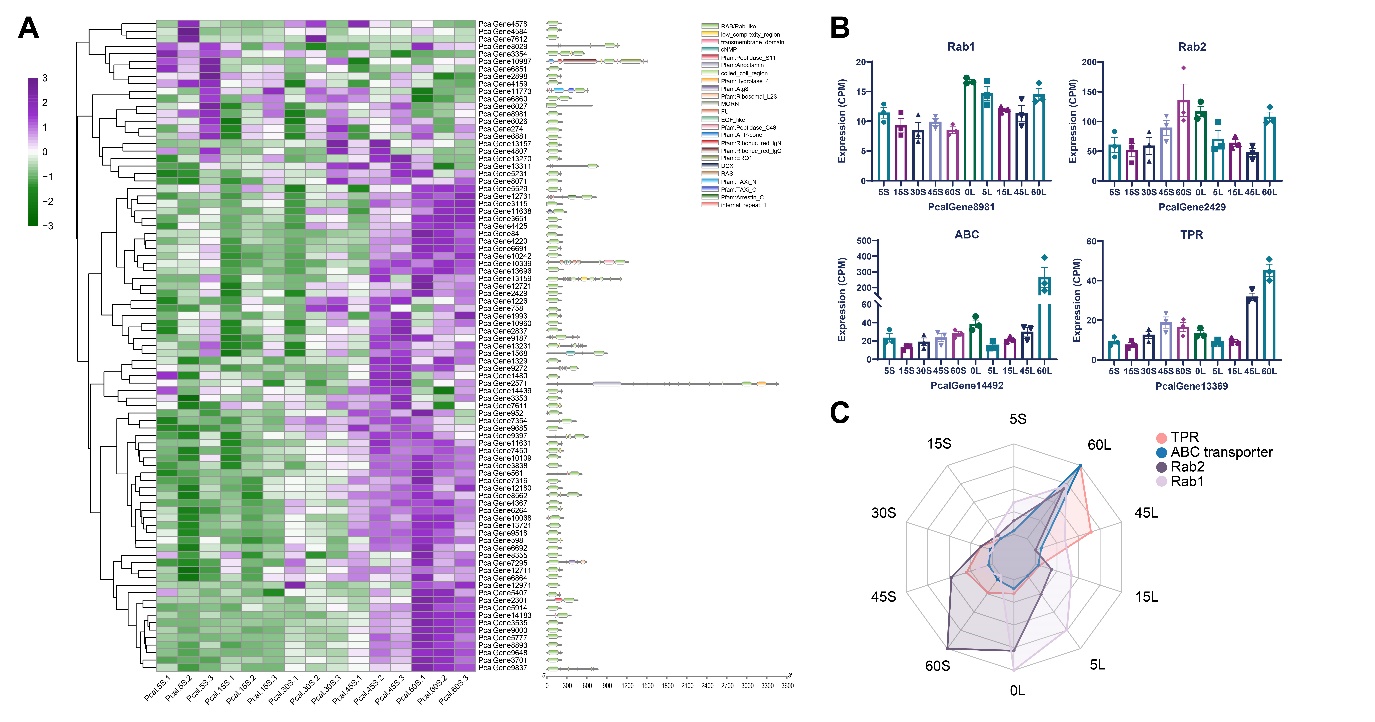
**

**Figure S6. Alternative splicing events under osmotic pressure, related to Figure 5.**

(A) The transcriptome profiles of Rab/Rab-like protein-coding genes upon short-term treatment, Conserved domains are denoted as rounded rectangles in different colors.

(B) Expression levels of the Rab1, Rab2, ABC, and TPR genes. (C) Radar chart showing the expression levels of the Rab1, Rab2, ABC, and TPR genes.

**Supplementary table 1.** Information of somatic genome assemblies of *Paramecium* species.

**Supplementary table 2.** Classification of RNA types, subtypes, and quantity statistics

**Supplementary table 3.** GO pathway enrichment analysis results of the DEGs under different osmotic pressures.

**Supplementary table 4.** GO pathway enrichment analysis results of WGCNA gene modules with increased expression under hyperosmotic or hypoosmotic pressures upon long-term treatment.

**Supplementary table 5.** Copy number variations (CNVs) and gene expression correlations under 5‰ and 45‰ osmotic pressures

**Supplementary table 6.** MCMC convergence diagnostics for Bayesian divergence time estimation.

**Supplementary table 7.** Information on relevant genes and the primers used.
